# Adoptive cell therapy in lung cancer: progress, challenges, and prospects

**DOI:** 10.1016/j.pccm.2026.08.002

**Published:** 2026-09-07

**Authors:** Jingyi Li, Chan Tong, Yongchang Chen, Bo Zhu

**Affiliations:** Institute of Cancer, Xinqiao Hospital, Army Medical University, Chongqing 400037, China

**Keywords:** Lung cancer, Immunotherapy, Adoptive cell therapy

## Abstract

Lung cancer is the leading cause of cancer-related deaths worldwide. Over the past decade, immunotherapy, represented by immune checkpoint inhibitors (ICIs) targeting programmed cell death protein-1/programmed cell death protein ligand-1 (PD-1/PD-L1) or cytotoxic T lymphocyte associated antigen-4 (CTLA-4), has revolutionized the treatment paradigm of lung cancer, reducing the risk of death by approximately 30%. However, approximately 70% of patients with advanced non-small cell lung cancer are either non-responsive to ICIs or initially respond to treatment but eventually experience disease progression due to acquired resistance. This subset of non-responsive or ultimately resistant patients is often characterized by complete exhaustion of endogenous anti-tumor T cells, which are unable to exert anti-tumor effects, thereby leading to ICI non-response or resistance. Correspondingly, the adoptive transfer of immune cells has shown promising efficacy in numerous clinical studies involving this patient population. Nevertheless, the Food and Drug Administration (FDA) has not yet approved any cell therapy strategy for lung cancer, primarily because of factors such as the hostile tumor microenvironment and T cell exhaustion. Herein, we summarize the current progress in cell therapy for lung cancer, analyze key challenges, and propose potential solutions.

## Introduction

Lung cancer is a malignant tumor with high incidence and mortality rates. Globally, approximately 2.2 million new cases and 1.8 million deaths from lung cancer occur every year, of which approximately 85% are non-small cell lung cancer (NSCLC) and 15% are small cell lung cancer (SCLC).^1^^,^^2^ Although traditional treatment modalities, such as surgery, chemotherapy, and radiotherapy, have improved patient prognosis to a certain extent, the 5-year survival rate of patients with advanced lung cancer remains <20%.^3^ Tumor recurrence and drug resistance remain urgent challenges to be addressed in clinical treatment. Over the past decade, immunotherapy, represented by immune checkpoint inhibitors (ICIs) targeting programmed cell death protein-1/programmed cell death protein ligand-1 (PD-1/PD-L1) or cytotoxic T lymphocyte associated antigen-4 (CTLA-4), has revolutionized the treatment landscape for lung cancer, increasing the 5-year survival rate of patients with advanced NSCLC to approximately 30%.^4^^,^^5^ Despite these remarkable achievements, PD-1/PD-L1 inhibitors still have limitations, such as limited treatment response rates and primary or secondary resistance. The status of tumor-infiltrating CD8⁺ T cells is crucial for the response to PD-1/PD-L1 antibody therapy.^6^^,^^7^ Single-cell sequencing results have shown that tumor-infiltrating CD8⁺ T cells in patients with NSCLC constitute a highly heterogeneous population.^6^^,^^7^ T cell transcription factor 1 (TCF-1)⁺ precursor exhausted CD8⁺ T cells are the key cell subset mediating the response to PD-1/PD-L1 antibody treatment.^8^ In contrast, the core mechanism underlying poor response or acquired resistance to ICIs in patients is often the profound exhaustion of tumor-infiltrating CD8⁺ T cells, accompanied by a significant reduction in the proportion of TCF-1⁺ precursor exhausted CD8⁺ T cells.^9^ When endogenous TCF-1⁺ precursor exhausted CD8⁺ T cells are depleted or dysfunctional, adoptive cell therapy (ACT) emerges as a promising strategy.

Immune cells that directly exert anti-tumor effects include CD8⁺ T and natural killer (NK) cells. Current adoptive T cell therapies include tumor-infiltrating lymphocyte (TIL) therapy, chimeric antigen receptor (CAR)-T cell immunotherapy, and T cell receptor (TCR)-engineered T cell (TCR-T) therapy. TIL therapy involves the adoptive transfer of autologous tumor-infiltrating lymphocytes from patients. TIL therapy yields an objective response rate (ORR) of approximately 21.4% in patients with advanced NSCLC.^10^ CAR-T and TCR-T cell therapies utilize genetic engineering approaches to modify peripheral blood T cells into tumor-reactive T cells. Specifically, CAR-T cell therapy enables T cells to express CAR constructs that recognize the extracellular domains of tumor antigens, thereby allowing tumor cell death in a major histocompatibility complex (MHC)-independent manner. Currently, CAR-T therapies targeting epidermal growth factor receptor (EGFR), human epidermal growth factor receptor-2 (HER2), mesothelin (MSLN), carcinoembryonic antigen (CEA), and other antigens are being explored in early-phase lung cancer trials.^11^^,^^12^ Unlike CARs, TCRs typically exhibit moderate antigen affinity and activate physiological signaling pathways, which may enhance T cell persistence and thus improve anti-tumor efficacy. Therefore, TCR-T therapy may have greater advantages for the treatment of solid tumors, such as better adaptation to the harsh solid tumor microenvironment (TME).^13^ TCR-T cells targeting New York esophageal squamous cell carcinoma-1 (NY-ESO-1) offer new hope for patients with advanced lung cancer. In a clinical case report published in *Oncology Letters*, a female patient with advanced lung adenocarcinoma, who was positive for both human leukocyte antigen (HLA)-A2 (HLA-A2), and NY-ESO-1, achieved a partial response (PR) lasting 4 months following adoptive immunotherapy with NY-ESO-1-targeted TCR-T cells.^14^ NK cell-based cytokine-induced killer (CIK) cell therapy and NK cell therapy have also achieved certain clinical breakthroughs. The combination regimen of CIK cell therapy with PD-1 inhibitors and chemotherapy significantly prolongs the progression-free survival (PFS) of patients with advanced NSCLC and shows a trend toward long-term survival benefits^15^; additionally, NK cell therapy has excellent potential in reversing EGFR-targeted drug resistance, substantially improving the response rate.^16^

Although lung cancer cell therapy is still in the clinical trial phase and the Food and Drug Administration (FDA) has not yet approved any cell therapy products for lung cancer, the available clinical data not only highlight the strategic value of immune cell therapy in the comprehensive treatment of lung cancer but also open up new avenues for overcoming the drug resistance in ICIs and targeted therapies. Despite the considerable clinical potential of ACT for lung cancer treatment, multiple challenges remain. For instance, the abnormal tumor vasculature and dense stroma in lung cancer not only restrict T cell infiltration into the tumor, but the immunosuppressive TME also impairs the persistent survival and functional activity of T cells. Furthermore, given the high heterogeneity of lung cancer, there is a significant variability in antigen expression among different tumor cells. Consequently, CAR-T or TCR-T therapies targeting a single antigen are prone to failure owing to the downregulation or loss of target antigens. In the future, as more clinical data accumulate and treatment strategies are optimized, cell therapy is expected to become an important component of precision immunotherapy systems for lung cancer, bringing hope to more patients. This review provides a comprehensive overview of the latest advances in lung cancer cell therapy, along with the challenges and corresponding strategies for future research and clinical applications.

## Current ACT in lung cancer

ACT involves the infusion of TILs or T cells derived from the peripheral blood into patients. The development of ACT began with the *ex vivo* expansion and adoptive transfer of TILs, a process that amplifies a heterogeneous population of endogenous T cells found within harvested tumors. Subsequently, ACT has evolved to utilize autologous T cells obtained from peripheral blood, which are activated *ex vivo* and genetically engineered to express transgenic antigen receptors, such as TCRs or CARs.^14^^,^^17^ CAR-T and TCR-T cell therapies require the expansion of genetically engineered T cells directed against specific antigenic targets. Currently, ACT has achieved significant success in treating refractory B cell malignancies, multiple myeloma, and melanoma.^18^, ^19^, ^20^ In contrast, the efficacy of ACT in solid tumors, such as lung cancer, remains limited,^21^^,^^22^ and its application in the context of lung cancer is still at an early stage of development. Below, we discuss ongoing research on ACT in solid tumors, especially in lung cancer.

### TIL therapy

Unlike hematological malignancies, lung cancer is comprised of a polyclonal cell population characterized by heterogeneous antigens. Consequently, TILs isolated from tumor tissue represent a heterogeneous population of polyclonal cells capable of recognizing a diverse array of tumor antigens.^23^^,^^24^ Autologous TIL therapy was first reported to possess potent anti-tumor activity in metastatic melanoma.^25^ To date, TIL therapy has made substantial progress in melanoma clinical research.^26^, ^27^, ^28^, ^29^ The most compelling results indicate that the ORR and complete response (CR) rate in patients with melanoma receiving TIL therapy are 40–50% and 20%, respectively, and that patients achieving CR show long-term disease-free survival.^26^ TIL therapy can also induce durable remission in a subset of patients with other solid tumors, such as cervical cancer,^30^ colorectal cancer,^31^ and breast cancer.^32^

In the earliest clinical study evaluating the efficacy of TIL therapy in NSCLC, all patients at pathological stage II, IIIa, or IIIb (according to the Mountain staging system) underwent surgical resection as primary treatment and received TIL therapy or standard adjuvant therapy. Patients in the experimental group had a median overall survival of 22.4 months, compared with 14.1 months for those receiving standard treatment.^33^ This study provides strong evidence supporting TIL therapy as a viable treatment option for patients with advanced lung cancer. In a study of patients with metastatic lung cancer resistant to anti-PD-1 therapy, 3 of 13 evaluable patients receiving TIL therapy achieved a confirmed response, and 11 showed a reduction in tumor burden. Notably, two patients achieved durable complete responses after 1.5 years.^34^ In a Phase I trial, 13 patients who progressed on nivolumab treatment achieved an ORR of 23% following autologous TIL therapy.^34^ A Phase II clinical trial is currently evaluating the efficacy of autologous TIL therapy in patients with solid tumors (NCT03645928).

Lifileucel is the first autologous TIL therapy approved for the treatment of solid tumors,^20^ specifically melanoma.^35^^,^^36^ Currently, research on its application in lung cancer has also made progress. In a study of NSCLC, lifileucel as monotherapy achieved an ORR of 21.4% in 28 patients who were resistant to ICIs.^10^ Table 1 summarizes other ongoing clinical trials utilizing TILs for the treatment of lung cancer.

**Table 1:** Selected clinical trials of TIL therapy in lung cancer.

NCT No.	Title	Phase	Type of cancer	State
NCT03215810	Nivolumab and TIL in advanced NSCLC	I	Stage IV or recurrent NSCLC	Completed
NCT05681780	Clinical trial of CD40L-augmented TIL for patients with *EGFR, ALK, ROS1* or *HER2*-driven NSCLC	I/II	Stage IV or recurrent NSCLC	Recruiting
NCT05878028	L-TIL plus tislelizumab for PD-1 antibody-resistant aNSCLC	II	Stage IV NSCLC	Recruiting
NCT04614103	Autologous LN-145 in patients with metastatic NSCLC	II	Metastatic stage IV NSCLC	Recruiting
NCT03419559	Study of autologous TILs (LN-145) in combination with durvalumab in NSCLC	II	Stage III or stage IV NSCLC	Withdrawn: An additional NSCLC arm added to the IOV-COM-202 study
NCT03645928	Study of autologous TILs in patients with solid tumors	II	Unresectable or metastatic melanoma; advanced, recurrent or metastatic HNSCC; stage III or stage IV NSCLC	Recruiting
NCT05566223	CISH-inactivated TILs in the treatment of NSCLC (CheckCell-2)	I/II	PD-L1 negative or positive metastatic NSCLC	Withdrawn: No recruitment
NCT05573035	A study to investigate LYL845 in adults with solid tumors	I	Melanoma, NSCLC, CRC	Terminated: Pipeline reprioritization
NCT05576077	A study of TBio-4101 (TIL) and pembrolizumab in patients with advanced solid tumors (STARLING)	I	Advanced or metastatic breast carcinoma, colorectal adenocarcinoma, uveal melanoma, cutaneous melanoma, NSCLC, HNSCC	Terminated: Sponsor decision
NCT02133196	T cell receptor immunotherapy for patients with metastatic NSCLC	II	Stage IV or unresectable NSCLC	Recruiting
NCT03903887	A study of anti-PD-1 antibody-activated TILs in NSCLC	I/II	Stages II–IIIA NSCLC	Unknown status: Study completed past its due date; status unverified for over 2 years
NCT05361174	A study to investigate the efficacy and safety of an infusion of IOV-4001 in adult participants with unresectable or metastatic melanoma or stage III or IV NSCLC	I/II	Unresectable or metastatic melanoma or stage III or IV NSCLC	Recruiting
NCT01820754	Evaluation of circulating T cells and TILs during/after pre-surgery chemotherapy in NSCLC	II	Stages 1B, 2, or 3 NSCLC	Completed
NCT06538012	TIL therapy combined with pembrolizumab for advanced or metastatic refractory lung cancer	I/II	Advanced or metastatic refractory lung cancer	Recruiting
NCT05902520	Adoptive cell therapy using cancer-specific CD8^+^ TILs in adult patients with solid tumors	I	HNSCC, melanoma, gynecologic cancer, CRC, lung cancer, urogenital cancer	Active, not recruiting
NCT06375187	A study of GC203 TIL in advanced malignant solid tumors (KUNLUN-001)	I	Solid tumor: gynecologic cancer, breast cancer, gastrointestinal cancer, lung cancer	Recruiting
NCT06455917	TIL Therapy in NSCLC (BaseTIL-02 L)	II	NSCLC	Recruiting
NCT06473961	A Phase Ib Study of GC101 in NSCLC (MIZAR-005)	I	Unresectable advanced, recurrent, or metastatic NSCLC	Recruiting
NCT06630611	Evaluation of a pragmatic approach to ACT using an IL-2 analog (ANV419) *vs*. high dose IL-2 after TIL therapy in patients with melanoma, NSCLC and cervical cancer (PragmaTIL) (PragmaTIL)	II	Histologically or cytologically proven metastatic or unresectable cutaneous melanoma, NSCLC, or cervical cancer	Recruiting
NCT06863233	A study of CD8^+^ T cell imaging during treatment in people with NSCLC	I	Metastatic NSCLC	Recruiting
NCT05397093	ITIL-306 in advanced solid tumors	I	High-grade serous epithelial carcinoma of the ovary, fallopian tube, or peritoneum, adenocarcinoma of the lung, or clear-cell renal cell carcinoma	Terminated: Discontinued development of the investigational program
NCT06107894	TIL therapy for patients with advanced solid tumors	I	Breast or lung cancer	Not yet recruiting
NCT06519669	Autologous tumor-infiltrating lymphocyte injection (GT201) for treatment of patients with advanced lung cancer	Not applicable	Late-stage NSCLC without driver genes that have failed first-line systemic treatment; SCLC with first-line systemic treatment failure	Terminated: Terminated from a business perspective
NCT05676749	C-TIL051 in NSCLC	I	Stage IV or recurrent NSCLC	Terminated: Sponsor decision
NCT06491225	Autologous TIL (GTE-001 injection) for treatment of patients with advanced lung adenocarcinoma	I	Lung adenocarcinoma	Terminated: terminated from a business perspective
NCT06237881	A phase 1/2 study of KSQ-001EX, autologous TILs engineered to inactivate the *SOCS1* gene, in patients with select advanced solid tumors	I/II	Melanoma; NSCLC	Active, not recruiting

*ALK*, Anaplastic lymphoma kinase; aNSCLC, Advanced non-small cell lung cancer; CD40L, CD40 ligand; CISH, Cytokine inducible SH2 containing protein; CRC, Colorectal cancer; *EGFR*, Epidermal growth factor receptor; *HER2*, Human epidermal growth factor receptor 2; HNSCC, Head and neck squamous cell carcinoma; L-TIL, Liquid tumor-infiltrating lymphocytes; NSCLC, Non-small cell lung cancer; PD-1, Programmed cell death protein-1; PD-L1, Programmed death ligand 1; *ROS1*, ROS proto-oncogene 1, receptor tyrosine kinase; SCLC, Small cell lung cancer; *SOCS1*, Suppressor of cytokine signaling 1; TIL, Tumor-infiltrating lymphocyte.

TIL therapy may offer distinct advantages in the treatment of solid tumors. As previously mentioned, TILs exhibit diverse TCR clonality, enabling the recognition of multiple tumor antigens. Therefore, compared with other ACT modalities (such as CAR-T and TCR-T cell therapies), TILs may be superior in addressing tumor heterogeneity. Consequently, TILs demonstrate better clinical efficacy in solid tumors with high mutational burdens (such as melanoma) than other ACT methods.^37^ However, TIL therapy faces significant challenges regarding reproducibility and scalability. First, TILs are derived from tumor tissue, which is a paradoxical approach for patients who are candidates for surgical resection or who have tumor recurrence. Furthermore, although the manufacturing success rate of TILs is between 80% and 90%, there remains a risk of treatment cancellation due to manufacturing defects.^23^ Similarly, the complex TME of solid tumors affects the therapeutic efficacy of TILs. Immunosuppressive signals within the TME and chronic TCR stimulation can induce T cell exhaustion and dysfunction, further limiting T cell potency and persistence.^18^ Moreover, TIL therapy can lead to various treatment-related adverse events (TRAEs), including fever, hypotension, hypoxia, diarrhea, and myelosuppression.^38^ Currently, the method for *in vitro* culture and expansion of TILs relies on interleukin (IL)-2; however, high doses of IL-2 can induce systemic toxicity^39^ and may also promote regulatory T cells that suppress the anti-tumor response of TILs.^40^

### TCR-T therapy

The TCR is a critical surface molecule that specifically recognizes antigens and mediates immune responses.^41^ TCR-T therapy employs genetic engineering techniques to express TCR gene sequences specific to tumor antigens in T cells, thereby endowing them with the ability to specifically kill tumor cells.^13^^,^^42^^,^^43^ TCR-T cells can recognize both surface antigens on tumor cells and intracellular antigens presented by specific MHC molecules, thereby possessing a broader capability for target antigen recognition. This not only enhances T cells’ ability to recognize tumor cells but also improves their persistence.^13^^,^^44^ Furthermore, TCR-T therapy utilizes peripheral lymphocytes, offering greater reproducibility and scalability than TIL technology.^44^ Currently, TCR-T cells targeting tumor-associated antigens (TAAs) or tumor-specific antigens (TSAs) have shown promising results in clinical trials for melanoma, colorectal cancer, synovial sarcoma, and esophageal cancer.^39^

Research on TCR-T cell therapy for lung cancer has also progressed in recent years. NY-ESO-1 is expressed in approximately 11.8–21% of NSCLC cases.^45^^,^^46^ A Phase I trial evaluated the safety and feasibility of NY-ESO-1 TCR-T cell therapy in four patients with metastatic NSCLC (mNSCLC) who were HLA-A2 positive (NCT02457650).^14^ Two patients had clinical responses to NY-ESO-1 TCR-T cell treatment, with one patient achieving a short-term PR within 4 months, and none of the four patients experienced severe toxicity. Phase I trials of NY-ESO-1 TCR-T cells used alone or in combination with pembrolizumab for advanced NSCLC are ongoing (NCT03029273 and NCT03709706). Another Phase I clinical trial evaluated the safety and efficacy of melanoma-associated antigen A10 (MAGE-A10)-specific TCR-T cells in patients with MAGE-A10^+^ advanced NSCLC.^47^ Among the 11 patients treated, 1 had a PR, 4 had stable disease, 5 had clinical or radiographic progressive disease, and 1 was not evaluable. Other clinical trials are currently underway to determine the feasibility, safety, and efficacy of this personalized therapy for NSCLC (Table 2).

**Table 2:** Selected clinical trials of TCR-T cell therapy in lung cancer.

NCT No.	Title	Phase	Type of cancer	Status
NCT03778814	TCR-engineered T (TCR-T) cell immunotherapy of lung cancer and other solid tumors	I	Advanced lung tumor or other solid tumor	Recruiting
NCT02592577	MAGE A10c796T for advanced non-small cell lung cancer (NSCLC)	I	Stage IIIb or stage IV NSCLC	Completed: No results posted
NCT05194735	Phases I/II study of autologous T cells to express TCRs in subjects with solid tumors	I/II	Gynecologic cancer, CRC, pancreatic cancer, NSCLC, cholangiocarcinoma	Not recruiting
NCT02588612	Letetresgene autoleucel engineered T cells in NY-ESO-1 positive advanced NSCLC	I	Stage IIIb or stage IV NSCLC	Completed
				
NCT04639245	Genetically engineered cells (MAGEA1-specific T cell receptor-transduced autologous T cells) and atezolizumab for the treatment of metastatic triple-negative breast cancer (TNBC), urothelial cancer, or NSCLC	I/II	MAGEA1 positive TNBC, urothelial carcinoma, or NSCLC	Completed
NCT06043713	Autologous CD8^+^ and CD4^+^ transgenic T cells expressing high-affinity *KRAS* G12V mutation-specific T cell receptors (FH-A11KRASG12V-TCR) in treating patients with metastatic pancreatic, colorectal, and non-small cell lung cancers with *KRAS* G12V mutations	I	Metastatic pancreatic, colorectal and NSCLC with *KRAS* G12V mutations	Recruiting
NCT03132922	MAGEA4c1032T for multi-tumor	I	MAGE A4 positive solid tumors	Active, not recruiting
NCT01967823	T cell receptor immunotherapy targeting NY-ESO-1 for patients with NY-ESO-1 expressing cancer	II	NY-ESO-1-expressing cancer	Completed
NCT05296564	Anti-NY-ESO-1 TCR gene-engineered lymphocytes given by infusion to patients with NY-ESO-1-expressing metastatic cancers	I/II	NY-ESO-1-expressing metastatic cancers	Recruiting
NCT06942143	An open-label, phase I clinical trial of super1 TCR-T in NY-ESO-1-positive patients with advanced solid tumors	I	NY-ESO-1-expressing sarcoma, lung cancer and melanoma	Recruiting
NCT06619886	TP53 R248Q TCR-T cell therapy for advanced solid tumor	I	Lung carcinoma, colorectal adenocarcinoma, pancreatic adenocarcinoma, any other solid tumor expressing the *TP53* R248Q mutation	Not yet recruiting
NCT06767046	KRAS-specific autologous TCR-T cell therapy for *KRAS* mutation in advanced solid tumors	I	Solid tumors (e.g., CRC, pancreatic cancer, NSCLC) with *KRAS* G12V mutations	Active, not recruiting
NCT05483491	KK-LC-1 TCR-T cell therapy for gastric, breast, cervical, and lung cancer	I	Metastatic solid tumor with ≥10% of tumor cells positive for KK-LC-1	Recruiting
NCT05124743	HLA typing & tumor neoantigen identification for Phase I/II study of autologous TCR-T cells in subjects with solid tumors	I/II	Gynecologic cancer (ovarian and endometrial), CRC, pancreatic cancer, NSCLC	Terminated: No results posted
NCT05035407	T cell receptor gene therapy targeting KK-LC-1 for gastric, breast, cervical, lung and other KK-LC-1 positive epithelial cancers	I	KK-LC-1 positive epithelial cancer	Terminated
NCT06218914	Phase 1 study to investigate TCRTs *KRAS* mutation in unresectable, advanced, and/or metastatic solid tumors	I	NSCLC, colorectal adenocarcinoma, pancreatic adenocarcinoma, endometrial cancer or any other solid tumor and *KRAS* G12D variant mutation	Recruiting
NCT03412877	Administration of autologous T cells genetically engineered to express T cell receptors reactive against neoantigens in people with metastatic cancer	II	Gastrointestinal and genitourinary cancers; breast, ovarian, and other solid cancers; NSCLC; endocrine tumors including neuroendocrine tumors and, multiple myeloma with solid masses (plasmacytomas)	Recruiting
NCT02869217	Study of TBI-1301 (NY-ESO-1 specific TCR gene-transduced autologous T lymphocytes) in patients with solid tumors	I	Metastatic or recurrent unresectable solid tumor with NY-ESO-1 expression	Active, not recruiting
NCT04526509	Master protocol to assess safety and dose of first-time in human next-generation engineered T cells in NY-ESO-1 and/or LAGE-1a positive advanced solid tumors	I	Stage IV NSCLC with NY-ESO-1 and/or LAGE-1a expression	Terminated
NCT06105021	Phase I study of autologous CD8^+^ and CD4^+^ engineered T cell receptor T cells in subjects with advanced or metastatic solid tumor	I	NSCLC with *KRAS* G12V mutation	Active, not recruiting
NCT02408016	Genetically modified T cells in treating patients with stage III–IV NSCLC or mesothelioma	I/II	Stage III–IV NSCLC or mesothelioma	Terminated

CRC, Colorectal cancer; KK-LC-1, Kita-Kyushu lung cancer antigen-1; KRAS, Kirsten rat sarcoma 2 viral oncogene homologue; HLA, Human leukocyte antigen; LAGE-1a, Lymphocyte-activating factor-1a; MAGE, Melanoma-associated antigen; MAGE A, Melanoma-associated antigen family A; NSCLC, Non-small cell lung cancer; NY-ESO-1, New York esophageal squamous cell carcinoma-1; TCR, T cell receptor; TCR-T, T cell receptor engineered T.

Afami-cel is a melanoma-associated antigen A4 (MAGE-A4)-targeting TCR-T cell therapy, the first and currently only FDA-approved TCR-engineered T cell therapy for the treatment of advanced synovial sarcoma.^48^^,^^49^ In the Phase I SURPASS trial, lete-cel monotherapy demonstrated responses in patients with refractory solid tumors, such as ovarian cancer, gastroesophageal junction cancer, urothelial carcinoma, head and neck cancer, and synovial sarcoma (although responses have not yet been observed in lung cancer).^50^ However, trials are already evaluating the efficacy of lete-cel, used alone or in combination with nivolumab or pembrolizumab (NCT04044859), including patients with NSCLC. Lete-cel consists of TCR-T cells that recognize a shared epitope of the cancer testis antigens NY-ESO-1 and L antigen family member 1 isoform A (LAGE-1a). A study evaluating the efficacy of lete-cel alone or in combination with pembrolizumab in patients with advanced NSCLC found that the toxicity profiles of both monotherapy and combination therapy were manageable, but anti-tumor activity was limited. More clinical studies on TCR-T therapy for lung cancer are ongoing and are summarized in Table 2.

The widespread clinical application of TCR-T cell therapy in solid tumors faces numerous challenges,^13^ such as the heterogeneity of intratumoral antigen expression in lung cancer and the immunosuppressive TME. TCR-T therapy targets antigens in an HLA-restricted manner. Therefore, patients receiving treatment require a match for both the HLA allele and target tumor antigen, which presents new challenges for the clinical application of TCR-T therapy.^18^

### CAR-T therapy

TCR-T and CAR-T therapies involve the genetic modification of T cells to express receptors that target tumor antigens. Unlike TCR-T cells, which rely on peptide–MHC complexes for tumor cell recognition, CAR-T therapy involves genetically engineering T cells to express CARs (which recognize specific tumor antigens in an MHC-independent manner).^51^, ^52^, ^53^ CARs are primarily composed of an extracellular antigen-recognition domain (ectodomain), a transmembrane domain, and an intracellular signaling domain (endodomain). The ectodomain, which determines the affinity of the CAR, includes a single-chain variable fragment (scFv) derived from an antibody, consisting of the antigen-binding regions composed of the heavy and light chains of a monoclonal antibody.^54^ CAR-T cells have garnered widespread attention due to their breakthrough success in hematological malignancies,^55^ with six FDA-approved therapies targeting CD19 or B-cell maturation antigen (BCMA). CAR-T cell therapy has also progressed in trials targeting lung cancer. Selection of surface antigens is crucial for CAR-T therapy. Currently, several CAR-T therapies targeting EGFR, HER2, PD-L1, CEA, prostate stem cell antigen (PSCA), Mucin 1 (MUC1), MSLN, glypican-3 (GPC3), and receptor tyrosine kinase-like orphan receptor 1 (ROR1) are being tested in Phase I trials, including those in patients with lung cancer.^11^^,^^12^^,^^56^

In a Phase I clinical study (NCT01869166), among 11 patients receiving EGFR-targeted CAR-T cell therapy, 2 achieved PR, and 5 had stable disease for 2–8 months; the treatment was well-tolerated with no severe toxicity.^57^ In a second trial (NCT03182816), among nine patients receiving EGFR-CAR-T cell therapy, one achieved a PR lasting over a year, six had stable disease, and two had progressive disease. The PFS for these nine patients was 7.13 months, and the median overall survival (OS) was 15.63 months.^58^ These two trials indicate that EGFR-CAR-T cell therapy is feasible and safe for treating patients with EGFR-positive NSCLC.

CEA is strongly expressed in lung cancer and is associated with a poor prognosis and metastasis. Therefore, clinical studies have evaluated the safety and efficacy of CEA CAR-T therapy in patients with CEA-positive lung cancer (NCT02349724 and NCT04348643). Similarly, MUC1 is considered a potential target for ACT, as it is highly expressed in lung cancer and promotes metastasis.^11^ CAR-T cells targeting MUC1 successfully eliminated NSCLC tumor cells^11^ and have been evaluated in lung cancer clinical trials (NCT03525782, NCT02587689, and NCT05239143).

Overexpression of the TAA MSLN is closely related to tumor invasiveness and is an attractive target in NSCLC.^59^ A Phase I study (NCT02414269) reported results of regionally delivered autologous MSLN-targeted CAR-T cell therapy for mesothelioma, metastatic lung cancer, and metastatic breast cancer. The results showed a median OS of 23.9 months (1-year OS rate of 83%) in patients receiving CAR-T cell infusion, with eight patients remaining stable for ≥6 months.^60^ Another Phase I/II clinical study of MUC1 CAR-T cells combined with *PDCD1* (encoding PD-1) knockout for the treatment of patients with advanced NSCLC is also underway (NCT03525782). Among the 20 patients participating in the study, 11 had stable disease, and 9 had progressive disease.^61^

Delta-like ligand 3 (DLL3), an inhibitory ligand for the Notch receptor, is expressed in the majority of SCLC tumors.^62^ A Phase I study of DLL3-targeted CAR-T cell therapy was conducted in patients with relapsed/refractory SCLC (NCT03392064). Among the four patients, one achieved PR, two had stable disease (SD), and one had PD. The median PFS was 3.7 months, and the median OS was 7.4 months (NCT03392064). This first-in-human trial suggests that CAR-T cell therapy is promising for the treatment of SCLC. According to ClinicalTrials.gov, more CAR-T cell therapies are currently undergoing Phase I or Phase II clinical trials for lung cancer; these trials are summarized in Table 3.

**Table 3:** Selected clinical trials of CAR-T cell therapy in lung cancer.

NCT No.	Title	Phase	Type of cancer	Status
NCT01869166	Treatment of chemotherapy refractory EGFR positive advanced solid tumors (CAR-T-EGFR)	I/II	Chemotherapy refractory EGFR-positive NSCLC	Unknown status: Study completed past its due date; status unverified for over 2 years
NCT02414269	Malignant pleural disease treated with autologous T cells genetically engineered to target the cancer-cell surface antigen mesothelin	I/II	NSCLC metastatic to the pleura previously	Active, not recruiting
NCT02587689	Phase I/II study of anti-Mucin1 (MUC1) CAR-T cells for patients with MUC1^+^ advanced refractory solid tumor	I/II	MUC1-positive NSCLC	Unknown status: Study completed past its due date; status unverified for over 2 years
NCT03525782	Anti-MUC1 CAR-T cells and PD-1 knockout engineered T cells for NSCLC	I/II	MUC1-positive NSCLC	Unknown status: Study completed past its due date; status unverified for over 2 years
NCT03356808	Antigen-specific T cells against lung cancer	I/II	Stage III, IV or relapsed lung cancer confirmed by histology and biopsy	Unknown status: Study completed past its due date; status unverified for over 2 years
NCT03198052	GPC3/mesothelin/Claudin18.2/GUCY2C/B7-H3/PSCA/PSMA/MUC1/TGFβ/HER2/Lewis-Y/AXL/EGFR-CAR-T cells against cancers	I	Lung cancer patients expressing GPC3/Mesothelin/Claudin18.2/GUCY2C/B7-H3/PSCA/PSMA/MUC1/TGFβ/HER2/Lewis-Y/AXL/EGFR protein	Recruiting
NCT02706392	Genetically modified T-cell therapy in treating patients with advanced ROR1^+^ malignancies	I	ROR1 ^+^ NSCLC	Terminated
NCT02349724	A clinical research of CAR-T cells targeting CEA positive cancer	I	Relapsed or refractory CEA positive lung cancer	Unknown status: Study completed past its due date; status unverified for over 2 years
NCT01935843	Treatment of chemotherapy refractory HER-2 positive advanced solid tumors	I/II	Chemotherapy refractory HER-2-positive NSCLC	Unknown status: Study completed past its due date; status unverified for over 2 years
NCT02713984	A clinical research of CAR-T cells targeting HER2 positive cancer	I/II	Relapsed or refractory HER2 positive lung cancer	Withdrawn: Reform CAR structure due to safety consideration
NCT03740256	Binary oncolytic adenovirus in combination with HER2-specific autologous CAR VST, advanced HER2 positive solid tumors	I	Advanced refractory HER2 positive solid tumor	Recruiting
NCT03330834	CAR-T cell immunotherapy for advanced lung cancer	I	Stages III B/IV NSCLC	Terminated
NCT06903117	Anti-CEA CAR-T for advanced CEA-positive lung carcinoma	I	CEA-positive advanced, metastatic or recurrent lung cancer, including NSCLC and SCLC	Recruiting
NCT06945523	Clinical study on chimeric antigen receptor T lymphocyte (CAR-T) targeting CEA for the treatment of CEA-positive advanced lung cancer	I	CEA-positive lung cancer, including NSCLC and SCLC	Recruiting
NCT06992583	Targeted anti-CEA CAR-T immunotherapy for advanced lung cancer	I	CEA-positive lung cancer, including NSCLC and SCLC	Recruiting
NCT06768151	A clinical trial of CEA targeting CAR-T for CEA-positive advanced lung cancer	I	CEA-positive lung cancer, including NSCLC and SCLC	Recruiting
NCT07179692	CEA-targeted CAR-T therapy in CEA-positive advanced solid tumors	I	CEA-positive solid tumors	Recruiting
NCT06006390	CEA targeting CAR-T in the treatment of CEA-positive advanced solid tumors	I/II	CEA-positive solid tumors	Recruiting
NCT06010862	Clinical study of CEA-targeted CAR-T therapy for CEA-positive advanced/metastatic malignant solid tumors	I	CEA-positive solid tumors	Recruiting
NCT07250386	A study of CEA-targeted CAR-T therapy in patients with CEA-positive advanced solid tumors (PBC102)	I	CEA-positive solid tumors	Recruiting
NCT07181720	CD70-targeted CAR-T therapy in CD70-positive advanced solid tumors	I	CD70-positive solid cancer	Recruiting
NCT06126406	Clinical study of CEA targeting chimeric antigen receptor T lymphocytes (CAR-T) for CEA positive advanced malignant solid tumors	I	CEA-positive solid tumors	Recruiting
NCT07116057	MOv19-BBz CAR-T cells in FRα^+^ cancers	I	FRa-expression NSCLC	Recruiting
NCT04348643	Safety and efficacy of CEA-targeted CAR-T therapy for relapsed/refractory CEA^+^ cancer	I/II	CEA-positive solid cancer	Unknown status: Study completed past its due date; status unverified for over 2 years
NCT04489862	αPD-1-MSLN—CAR-T cells for the treatment of MSLN-positive advanced solid tumors	Early I	MSLN-positive and PD-L1-positive solid cancer	Unknown status: Study completed past its due date; status unverified for over 2 years
NCT06885697	Anti-mesothelin TNaive/SCM hYP218 (TNhYP218) CAR-T cells in participants with mesothelin-expressing solid tumors including mesothelioma	I	MSLN-positive solid cancer	Recruiting
NCT03198546	GPC3-CAR-T cells for immunotherapy of cancer with GPC3 expression	I	Lung squamous carcinoma or hepatocellular carcinoma with the GPC3 expression	Recruiting

				
NCT07249879	This study is an open-label, phase I study to evaluate the safety, feasibility, cytokinetics, and preliminary efficacy of GC511B in DLL3^+^ relapsed/refractory small cell lung cancer	I	DLL3-positive relapsed/refractory SCLC	Recruiting
NCT06043466	A clinical trial targeting CEA chimeric antigen receptor T (CAR-T) for CEA positive advanced malignant solid tumors	I	CEA-positive advanced malignant solid tumors	Recruiting
NCT05060796	Study of CXCR5 modified EGFR targeted CAR-T cells for advanced NSCLC	Early I	EGFR-positive NSCLC	Recruiting
NCT05341492	EGFR/B7-H3 CAR-T on lung cancer and triple negative breast cancer	Early I	Patients with EGFR/B7-H3-positive advanced solid tumors (lung and triple-negative breast cancer)	Recruiting
NCT06196294	GPC3/mesothelin-CAR-γδT Cells against cancers	I	Patients with advanced cancer that expresses GPC3 or mesothelin protein	Recruiting
NCT06072989	Repeat intravenous infusions of B4T2-001 CAR-T without lymphodepleting chemotherapy for solid tumors	I	BT-001-positive solid tumors	Recruiting
NCT04684459	Dual-targeting HER2 and PD-L1 CAR-T for solid tumors	Early I	HER2-positive solid tumors	Recruiting
NCT06937567	CDH17 CAR-T therapy in advanced malignant solid tumors	Early I	CDH17-positive solid tumors	Recruiting
NCT05621486	A clinical study to evaluate B4T2-001 CAR-T cells in the treatment of advanced solid tumors	I	BT-001-positive malignant solid tumors	Active, not recruiting
NCT06051695	A study to evaluate the safety and efficacy of mesothelin-targeting logic-gated CAR-T, in participants with solid tumors that express MSLN and have lost HLA-A*02 expression (EVEREST-2)	I/II	MSLN expressing solid tumors	Recruiting
NCT05239143	P-MUC1C-ALLO1 allogeneic CAR-T cells in the treatment of subjects with advanced or metastatic solid tumors	I	Epithelial-derived cancer including NSCLC	Active, not recruiting
NCT05736731	A study to evaluate the safety and efficacy of A2B530, a logic-gated CAR-T, in participants with solid tumors that express CEA and have lost HLA-A*02 expression (EVEREST-1)	I/II	CEA-positive NSCLC	Active, not recruiting
NCT06682793	A study to evaluate the safety and efficacy of A2B395, an allogeneic logic-gated CAR-T, in participants with solid tumors that express EGFR and have lost HLA-A*02 expression (DENALI-1)	I/II	EGFR-positive NSCLC	Recruiting
NCT01583686	CAR-T cell receptor immunotherapy targeting mesothelin for patients with metastatic cancer	I/II	MSLN-expressing solid tumors	Terminated
NCT05680922	DLL3-directed chimeric antigen receptor T-cells in subjects with extensive stage small cell lung cancer	I	Unresectable SCLC, LCNEC, combined SCLC, or combined LCNEC	Active, not recruiting
NCT02862028	PD-1 antibody expressing CAR-T cells for EGFR family member positive advanced solid tumor (lung, liver and stomach)	I/II	EGFR family (including EGFR, HER2, and HER4)-positive solid tumors	Unknown status: Study completed past its due date; status unverified for over 2 years
NCT04025216	A study of CAR-T-TnMUC1 in patients with TnMUC1-positive advanced cancers	I	TnMUC1-positive NSCLC	Terminated: No results posted
NCT05620342	Autologous CAR-T cells targeting the GD2 antigen for lung cancer	Early I	Extensive stage SCLC or stage IV NSCLC	Recruiting
NCT03054298	CAR-T cells in mesothelin-expressing cancers	I	Metastatic or recurrent lung adenocarcinoma with documented pleural effusion	Completed
NCT07246304	TC-D101 cell therapy for patients with DLL3-positive SCLC	Early I	DLL3-positive relapsed/refractory SCLC	Recruiting
NCT06348797	Phase I clinical study of α-PD-L1/DLL3 CAR-T in patients with recurrent or refractory SCLC	I	Recurrent or refractory SCLC	Recruiting
NCT06972576	Clinical study of combined EphA2-targeted CAR-DC and CAR-T cell therapy for non-small cell lung cancer	I	EphA2-positive NSCLC	Recruiting
NCT04660929	CAR-macrophages for the treatment of HER2 overexpressing solid tumors	I	HER2-positive recurrent or metastatic solid tumors	Unknown status: Study completed past its due date; status unverified for over 2 years

α-PD-L1, Anti-programmed death-ligand 1 antibody; B7-H3, B7 homolog 3; CAR-DC, Chimeric antigen receptor-dendritic cell; CAR-T, Chimeric antigen receptor T cell; CDH17, Cadherin-17; CEA, Carcinoembryonic antigen; CTLA-4, Cytotoxic T-lymphocyte-associated antigen 4; CXCR5, C-X-C chemokine receptor type 5; DLL3, Delta-like ligand 3; EGFR, Epidermal growth factor receptor; EphA2, Ephrin type-A receptor 2; FRα, Folate receptor alpha; GUCY2C, Guanylate cyclase 2C; GD2, Disialoganglioside; GPC3, Glypican-3; HER2, Human epidermal growth factor receptor 2; HLA, Human leukocyte antigen; IGFR1: Insulin-like growth factor receptor 1; LCNEC, Large cell neuroendocrine lung carcinoma; MSLN, Mesothelin; MUC1, Mucin1; NSCLC, Non-small cell lung cancer; PD-1, Programmed cell death protein-1; PD-L1, Programmed death ligand 1; PSCA, Prostate stem cell antigen; PSMA, Prostate-specific membrane antigen; ROR1, Receptor tyrosine kinase-like orphan receptor 1; SCLC, Small cell lung cancer; TGFβ, Transforming growth factor-β; TnMUC1, Tn glycoform of mucin 1; VST, Virus-specific T cells.

The challenges facing CAR-T cell therapy are similar to those of TCR-T cell therapy, such as highly heterogeneous tumor antigen expression, an immunosuppressive microenvironment, tumor antigen escape, and the risk of on-target, off-tumor toxicity.^63^^,^^64^ Simultaneously, finding ideal TSAs or TAAs is crucial, as this remains the most direct and effective method for eliminating adverse effects on non-tumor cells and reversing the state of immune tolerance within the TME.

To facilitate cross-platform interpretation, Table 4 summarizes the published efficacies of TIL, TCR-T, and CAR-T therapies across lung cancer subtypes and treatment-line settings. This highlights the currently available differences in ORR and disease control rate (DCR), together with exploratory statistical comparisons, while underscoring the present evidence gaps in first-line disease and SCLC (Table 4). The NCT numbers of the clinical trials used for analysis, together with their original data, are presented in Supplementary Table 1.

**Table 4:** Comparison of published adoptive T cell therapy efficacy across lung cancer subtypes and treatment lines.

Subtype/treatment line/Indicator	ACT			Statistical comparison	*P*-value
	TIL	TCR-T	CAR-T		
NSCLC/first-line/ORR	10/22 (45.5%)	No published data	No published data	Not feasible	NA
NSCLC/later-line/ORR	19/80 (23.8%)	4/43 (9.3%)	4/48 (8.3%)	Fisher’s exact test across 3 ACT types	0.07
NSCLC/later-line/DCR	46/67 (68.7%)	6/16 (37.5%)	25/40 (62.5%)	Fisher’s exact test across 3 ACT types	0.063
SCLC/first-line/ORR	No published data	No published data	No published data	Not feasible	NA
SCLC/later-line/ORR	No published data	No published data	1/4 (25.0%)	Not feasible	NA
SCLC/later-line/DCR	No published data	No published data	3/4 (75.0%)	Not feasible	NA

ACT, Adoptive cell therapy; CAR-T, Chimeric antigen receptor T cell; DCR, Disease control rate; NA, Not applicable; NSCLC, Non-small cell lung cancer; ORR, Objective response rate; SCLC, Small cell lung cancer; TCR-T, T-cell receptor-engineered T cell; TIL, Tumor-infiltrating lymphocyte.

To further clarify the distinct translational features of major ACT platforms in lung cancer, Table 5 summarizes the main advantages, limitations, applicable clinical scenarios, and key research challenges of TIL, TCR-T, and CAR-T therapies. This overview is intended to help readers rapidly understand the relative strengths, current niches, and major barriers of each treatment strategy in lung cancer.

**Table 5:** Comparative overview of TIL, TCR-T, and CAR-T therapies in lung cancer: advantages, limitations, applicable scenarios, and major research challenges.

ACT modality	Key advantages	Key limitations	Most applicable scenarios in lung cancer	Major research challenges/bottlenecks
TIL therapy	Polyclonal tumor recognition; does not rely on a single predefined antigen; better suited to intratumoral heterogeneity; relatively encouraging clinical activity in NSCLC, especially after ICI resistance	Requires tumor tissue acquisition; individualized manufacturing is time-consuming and labor-intensive; product quality and expansion success may vary; T cells are still susceptible to exhaustion in the TME; IL-2-based expansion may cause toxicity	Advanced or metastatic NSCLC, particularly in patients with prior ICI exposure or acquired resistance; potentially more suitable for tumors with higher immunogenicity and broader endogenous T-cell reactivity	Difficulty obtaining adequate tumor tissue; limited scalability and reproducibility; manufacturing delay; variable TIL quality/function; immunosuppressive TME and T-cell exhaustion; currently very limited data in SCLC
TCR-T therapy	Can target intracellular antigens through peptide–MHC recognition; higher target specificity than TILs; more scalable and reproducible than TIL therapy because peripheral blood T cells are used; well suited for biomarker-driven precision therapy	Restricted by HLA type and antigen expression; only a subset of patients is eligible; vulnerable to antigen loss and impaired antigen presentation; efficacy may be limited in highly heterogeneous tumors; off-target/cross-reactivity remains a concern	Biomarker-selected NSCLC, especially tumors expressing shared cancer-testis antigens or mutation-derived neoantigens (e.g., NY-ESO-1, MAGE, KRAS, and WT1); suitable when a defined target and matched HLA background are present	Dual requirement for target antigen expression and HLA matching; narrow treatable population; antigen heterogeneity and immune escape; limited lung cancer-specific efficacy data; difficulty identifying optimal targets with sufficient tumor specificity
CAR-T therapy	MHC-independent recognition; flexible receptor engineering; broad target exploration in lung cancer; strongest pipeline expansion among ACT platforms; particularly attractive for surface antigens and currently the most active ACT strategy in SCLC (e.g., DLL3-directed approaches)	Limited to surface antigens; target heterogeneity and antigen escape are major barriers; poor trafficking, infiltration, and persistence in solid tumors; risk of on-target, off-tumor toxicity; clinical efficacy in lung cancer remains modest to date	Antigen-positive NSCLC (e.g., EGFR, HER2, MUC1, mesothelin, CEA) and SCLC, especially DLL3-positive relapsed/refractory disease; appropriate when a tumor-enriched surface antigen is identifiable	Lack of ideal lung cancer-specific surface targets; poor penetration into solid tumor tissue; suppressive TME; inadequate persistence; antigen escape; balancing efficacy with safety remains challenging, especially for broadly expressed TAAs

ACT: Adoptive cell therapy; CAR-T: Chimeric antigen receptor T cell; CEA: Carcinoembryonic antigen; DLL3: Delta-like ligand 3; EGFR: Epidermal growth factor receptor; HER2: Human Epidermal Growth Factor Receptor-2; HLA: Human leukocyte antigen; ICI: Immune checkpoint inhibitor; IL-2, Interleukin 2; MAGE: Melanoma-associated antigen; MHC: Major histocompatibility complex; MUC1: Mucin1; NSCLC: Non-small cell lung cancer; NY-ESO-1: New York esophageal squamous cell carcinoma-1; SCLC: Small cell lung cancer; TAA: Tumor-associated antigen; TCR-T: T-cell receptor-engineered T cell, TIL: Tumor-infiltrating lymphocyte; TME: Tumor microenvironment; WT1: Wilms tumor 1.

## Strategies to overcome the obstacles of ACT in lung cancer

There are a few barriers that hinder ACT from achieving therapeutic efficacy as satisfactory as that in lymphoma and multiple myeloma. As the most widely accepted subclasses in ACT, TIL, TCR-T, and CAR-T are faced with many challenges; each faces specific obstacles. With the development of gene-editing techniques, other immune cells, including NK cells and macrophages, have broadened the scope of engineered cellular immunotherapies for cancer. In addition, an accurate elucidation of the tumor immune microenvironment provides a glimpse toward personalized cellular therapy.

### Strengthening adoptive cells through construction modification

In contrast to hematological malignancies, the more condensed structure and complex cellular components, along with continuous antigenic stimulation, in lung cancer hamper the transfer of adoptive cells, leading to T cell exhaustion and shortened persistence. From the perspective of the basic CAR structure, Chen et al^65^ showed that positively charged patches (PCPs) on the surface of CAR mediate tonic signaling, which disturbs CAR-T efficacy, even before tumor antigen stimulation. In addition to the antigen-recognition domain, costimulatory signals are important for the activation and maintenance of transferred T cells. Currently, CD28 and 4-1BB (CD137) are the most commonly used cytoplasmic signaling domains in CAR-T cells, and both costimulation domains contribute to CAR-T cell persistence and cytotoxicity.^66^ The CD3 domain is also an indispensable structure in T cell activation and CAR signal transduction. Among the four subunits (ε, δ, γ, and ζ), CD3ζ directly conducts the final step of T cell receptor signal transduction from the membrane and thus is acknowledged by the US FDA as the standard intracellular activation domain in addition to costimulatory domains in CAR structure. However, other subunits have also been studied in recent years,^67^^,^^68^ revealing that CARs containing CD3ε, CD3δ, and CD3γ outperformed the traditional CD3ζ CAR-T cells. Thus, a more precise CAR molecular design is worth exploring and manipulating for the treatment of lung cancer^69^ (Fig. 1).

**Fig. 1:**
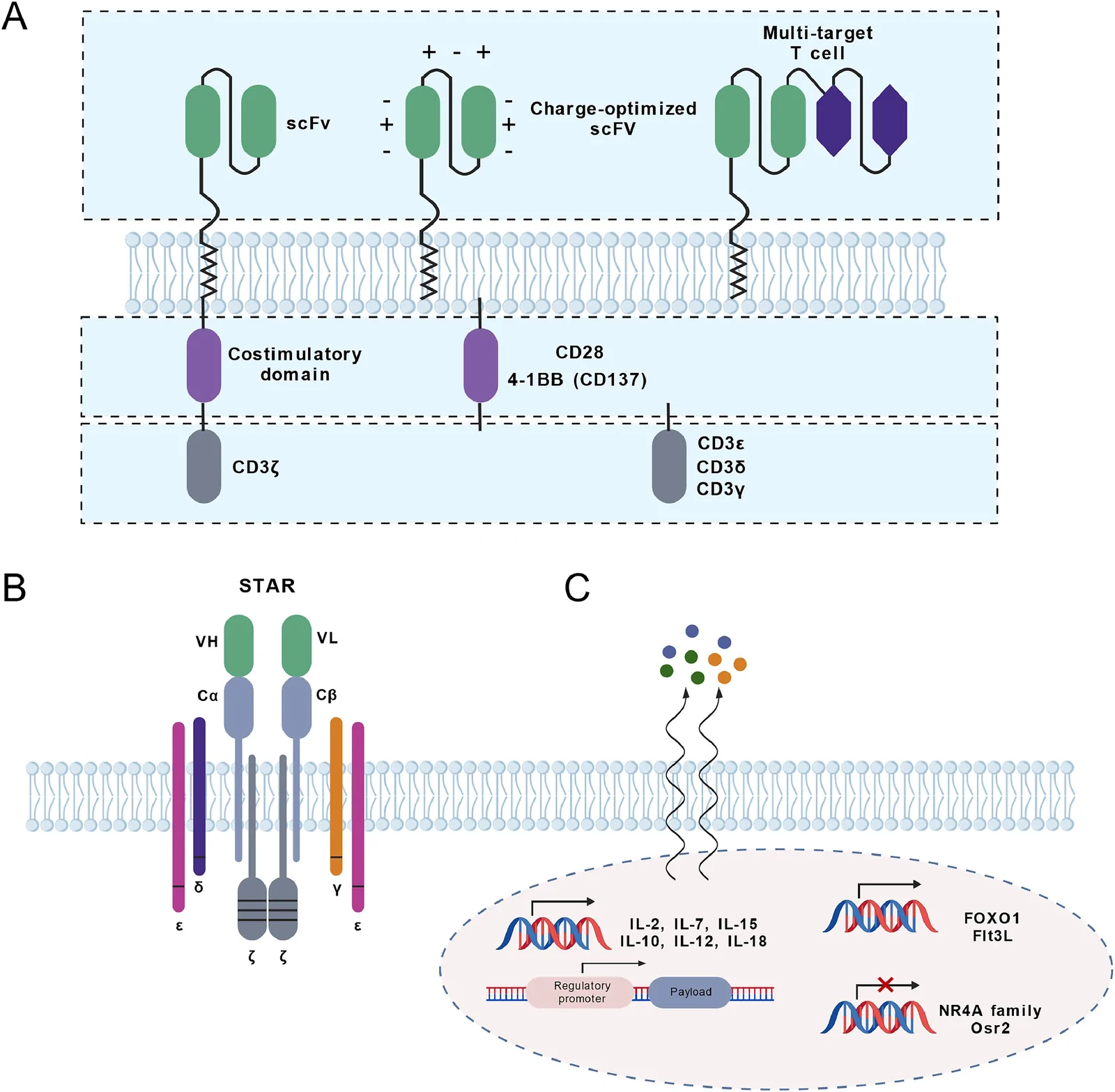
Candidate gene-editing methods to strengthen adoptive cells. (A) Basic CAR structure and specific domains under research. (B) Structure of STAR. (C) Candidate cytokines and transcription factors engineered in adoptive cells. Created with BioGDP.com. CAR, Chimeric antigen receptor; Flt3L, FMS-like tyrosine kinase 3 ligand; FOXO1, Forkhead box O1; IL, Interleukin; NR4A, Nuclear receptor subfamily 4 group A; Osr2, Odd-skipped related transcription factor 2; scFV, Single-chain variable fragment; STAR, Synthetic T cell receptor and antigen receptor; VH, Variable domain of the heavy chain; VL, Variable domain of the light chain.

It’s worth noting that some researchers believed that the artificial signaling domain on CAR molecules could not fully realize the potency of T cells.^70^ On this basis, Huang et al^71^ combined natural TCR signaling with endogenous CD3 signaling machinery and antigen-recognition domain from antibodies, constructing double-chain chimeric antigen receptor T cells, termed synthetic T cell receptor and antigen receptor T (STAR-T) cells (Fig. 1). STAR-T cells are engineered T cells that combine antibody-based tumor recognition with the natural T-cell receptor signaling system. Like CAR-T cells, STAR-T cells are designed to recognize tumor cells, but activate T cells in a more physiological manner, which may improve their sensitivity, persistence, and anti-tumor activity in solid tumors. STAR-T cells outperformed traditional BBz CAR-T (CAR-T with 4-1BB fused to the CD3ζ signaling chain) and 28z (CAR-T with CD28 fused to the CD3ζ signaling chain) CAR-T cells in proliferation, antigen sensitivity, and anti-tumor effects.^72^^,^^73^

In addition to CAR design, fast-growing omics and genetic engineering technologies have paved the way for decoding and manipulating gene expression to strengthen the anti-tumor efficiency of adoptive cells. The overexpression of forkhead box protein O1 (FOXO1)^72^^,^^73^ promotes a stem-like phenotype, thereby aiding the long-term persistence and clinical efficacy of therapeutic T cells. By assessing tumor-infiltrating T cells expressing the inhibitory molecules PD-1 and T cell immunoglobulin and mucin domain-containing protein 3 (Tim3), the nuclear receptor subfamily 4 group A (NR4A) family has been shown to be a key transcription factor that limits T cell function in solid tumors.^74^^,^^75^ Apart from the inhibitory chemical microenvironment, alterations in extracellular matrix (ECM) stiffness also impede T cell function. Osr2,^76^ which integrates biomechanical signaling, is selectively expressed in terminally exhausted CD8^+^ T cells, and could serve as a target for potentiating anti-tumor immunity in adoptive cells. In addition to modulating translational factors, another promising approach to address T cell dysfunction in tumors is engineering T cells to express immunomodulatory factors, including IL-2,^77^ IL-7,^78^^,^^79^ IL-15,^80^ IL-10,^81^^,^^82^ IL-12,^83^ IL-18,^84^ and Fms-like tyrosine kinase 3 ligand (Flt3L).^85^ As the core organs of gas exchange, the lungs possess a particular structure and cellular composition that are directly connected to the outside air. Given organ-specific and carcinogenic factors, elucidating the characteristics of antigen-specific T cells in the lung and the translational differences among their subclusters is urgently needed. Based on the currently investigated targets (including EGFR, Her2, PD-L1, CEA, PSCA, MUC1, MSLN, GPC3, and ROR1^12^), the structure of the antigen-recognition and signal transduction molecule and the regulation of gene expression in adoptive cells require careful matching and evaluation (Fig. 1).

Engineering T-cell activation signals represents an important strategy to optimize the efficacy and persistence of engineered cell therapies. According to the three-signal model of T-cell activation, Signal 1 is initiated by antigen recognition through the TCR or CAR, where optimized TCR/CD3 components, as mentioned above, can modulate proximal signaling intensity and functional output. Signal 2 is provided by co-stimulatory domains, in which CD28 predominantly promotes rapid proliferation through phosphoinositide 3-kinase (PI3K)–AKT–mammalian target of rapamycin (mTOR) signaling, whereas 4-1BB enhances mitochondrial fitness, memory formation and persistence through tumor necrosis factor receptor associated factor (TRAF)-mediated nuclear factor kappa-light-chain-enhancer of activated B cells (NF-κB) activation. Signal 3 is mediated by cytokine signaling and determines T-cell fate through Janus kinase (JAK)/signal transducer and activator of transcription (STAT)-dependent transcriptional programs. Therefore, rational manipulation of the three activation signals provides a comprehensive framework for next-generation cellular therapy engineering (Fig. 2).

**Fig. 2:**
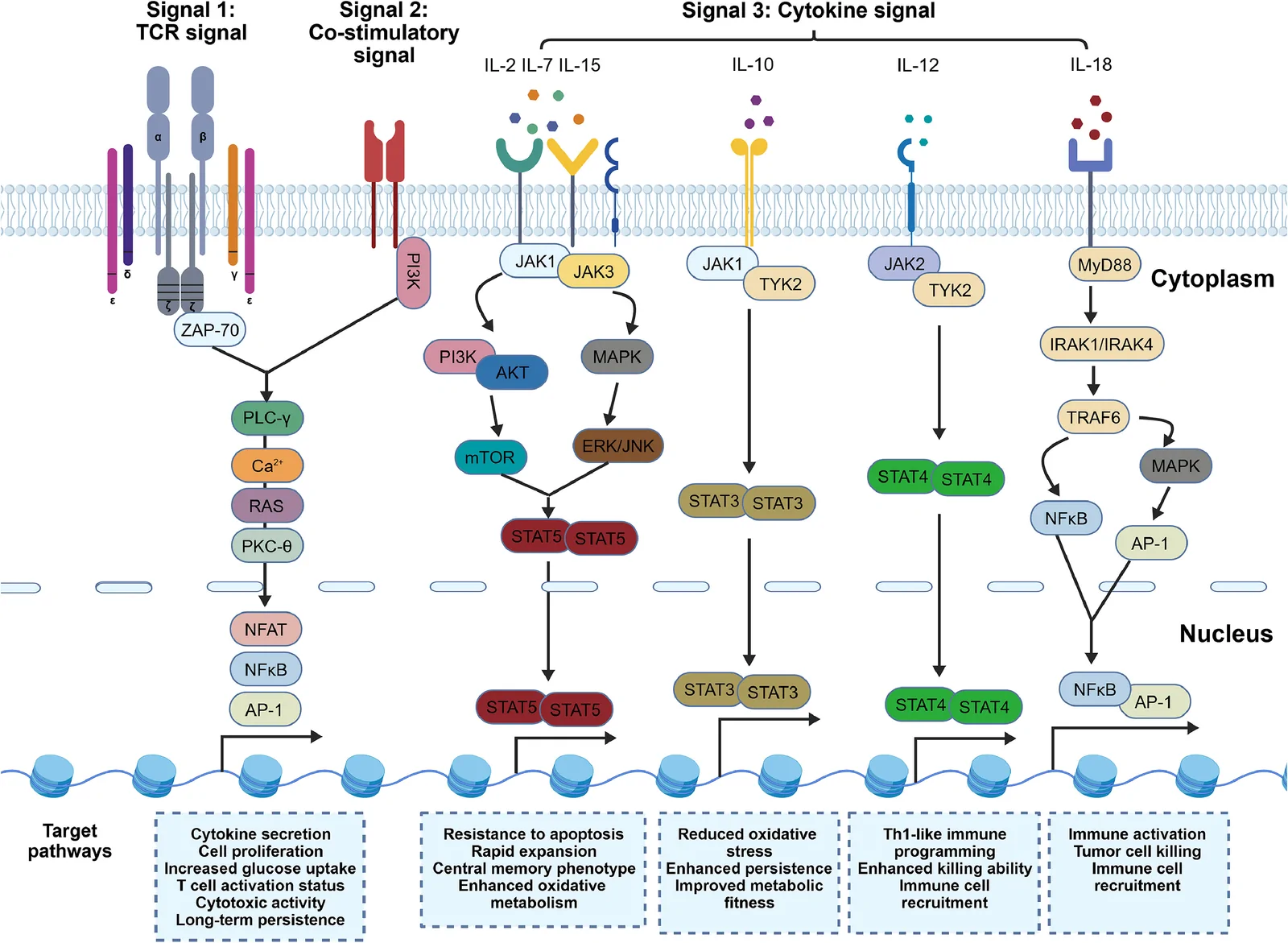
Targetable pathways based on the three signals of T cell activation for cell therapy. Created with BioGDP.com. AP-1, Activator protein 1; ERK, Extracellular signal-regulated kinase; IL, Interleukin; IRAK, Interleukin-1 receptor associated kinase; JAK, Janus kinase; JNK, c-Jun N-terminal kinase; MAPK, Mitogen-activated protein kinase; mTOR, Mammalian target of rapamycin; MyD88, Myeloid differentiation primary response 88; NFAT, Nuclear factor of activated T-cells; NFκB, Nuclear factor kappa-light-chain-enhancer of activated B cells; PI3K, Phosphoinositide 3-kinase; PKC-θ, Protein kinase C-θ; PLC-γ, Phospholipase C-gamma; STAT, Signal transducer and activator of transcription; TCR, T cell receptor; Th1, T helper 1; TRAF6, Tumor necrosis factor receptor associated factor 6; TYK2, Tyrosine kinase 2; ZAP-70, Zeta-chain-associated protein kinase 70 kDa.

### Balancing efficiency and security

The adverse effects of ACT limit the full expression of its potent anti-tumor activity. A large number of clinical studies have been terminated because of unexpected toxicities. As we have elaborated on ways to strengthen the potency of adoptive cells, it is also of great concern to restrict the expression of the armoring transgene to the tumor site to avoid unwanted damage to normal tissue. As early as a dozen years ago, researchers from Maryland^83^ took advantage of the natural T cell activation pathway, restricting the expression of armored IL-12 by placing its gene under the control of an artificial nuclear factor of activated T cells (NFAT) promoter. Lim, from the University of California, San Francisco, developed synNotch T cells.^77^^,^^86^^,^^87^ SynNotch T cells are synthetic receptor-engineered T cells in which recognition of an initial antigen triggers the programmed expression of a second therapeutic module, such as a CAR, cytokine, or other payload. This “sense-and-respond” design can improve tumor selectivity and reduce off-tumor toxicity by restricting T-cell activation to specific tissue or tumor contexts. Simic et al^88^ have already applied this technology in treating diseases of the central nervous system. Based on the identified extracellular central nervous system (CNS)-specific antigens, synthetic Notch receptors could induce the expression of diverse payloads only in the brain. This research may have great value as a reference for the ACT of lung cancer. Based on the methods applied in this study, molecular features unique to the lung extracellular environment are also worth considering and could serve as a paving stone for lung cancer-specific payload delivery following T cell transfer. On the other hand, a team from Peter MacCallum Cancer Centre^89^ challenged the traditional transgene-induction approaches. Using the clustered regularly interspaced short palindromic repeats (CRISPR) knock-in strategy, they leveraged the regulatory mechanisms of endogenous genes to drive transgene expression in a tumor-localized manner (compared to the spleen). The work mentioned above has prompted the development of a more precise and highly effective ACT that relies on organ-specific markers as triggers.

For CAR-T and TCR-T therapies, it is of great importance to determine the target antigen for the CAR or TCR molecules, as toxicity may arise from “on-target, off-tumor” effects. A prime example is a CAR-T clinical trial targeting the HER2 antigen. Since normal lung tissue vascular endothelial cells express low levels of HER2, the trial triggered rapid and fatal pulmonary toxicity in patients, ultimately leading to its termination.^57^ Unfortunately, lung cancer is known for its antigen heterogeneity.^18^ Among the tumor antigens in lung cancer, TAAs constitute the majority, whereas TSAs are rare. From this perspective, multi-target T cells (Fig. 1) have the potential to reduce “on-target” cytotoxicity in normal tissues through an elaborate design of the Boolean logic component. In 2024, a phase 1 trial^90^ reported reductions in tumor size in patients with recurrent glioblastoma after receiving treatment with bivalent CAR-T cells targeting EGFR and interleukin-13 receptor alpha 2 (IL13Rα2). Furthermore, the identification of tumor neoantigens is accelerating due to the rapid development of omics technologies and artificial intelligence.^91^ Therefore, results from clinical trials and advances in scientific techniques make it reasonable for us to anticipate more precisely targeted adoptive cells for lung cancer.

### Combination with other therapies

Over the past few decades, ICIs targeting the PD-1-PD-L1 axis or CTLA4 have revolutionized the therapeutic landscape in lung cancer.^18^ In the context of malignancy, tumor-specific T cells undergo activation-induced upregulation of coinhibitory receptors, which, in turn, suppress their activity. As adoptive cells are highly tumor antigen-specific, ICIs hold promise to fully realize their potency.^92^ There are already trials evaluating the safety and efficacy of combining CAR-T therapy with pembrolizumab in malignant pleural diseases.^60^^,^^93^ Cherkassky et al^93^ developed an anti-MSLN CAR with a dominant negative receptor of PD-1, which would compete for PD-L1/PD-L2 with endogenous PD-1 and enhance CAR-T cell anti-tumor ability. In addition, deletion of CTLA4 restores CAR-T cytotoxicity and rescues T cell function in patients resistant to CAR-T cell therapy.^94^ As an elaborately engineered genetic product, the adoptive cell can also serve as a carrier for the ICI. Rafiq et al^95^ modified CAR-T cells to secrete PD-1-blocking scFV. This method has also been tested in clinical trials for EGFR-positive advanced solid malignancies, including lung cancer (NCT02862028) (Fig. 3).

**Fig. 3:**
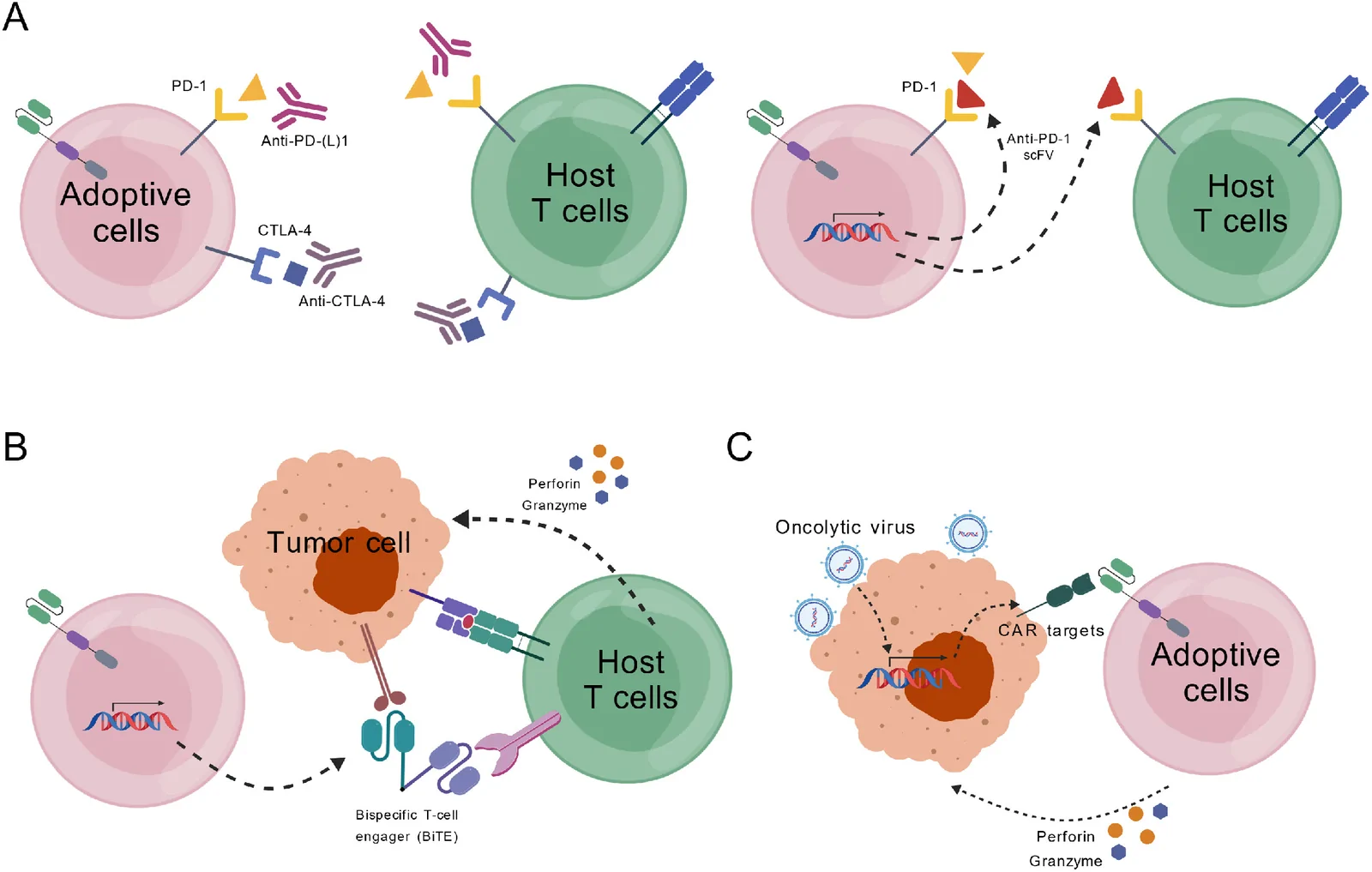
Other immune therapies that could be used in combination with adoptive cell therapy. (A) Adoptive cell therapy combined with immune checkpoint blockade therapy. (B) Adoptive cells engineered to express BiTE and facilitate host T cells to recognize and kill tumor cells. (C) Oncolytic viruses induce tumor cells to express target genes and facilitate adoptive cell therapy. Created with BioGDP.com. BiTE, Bispecific T cell engager; CAR, Chimeric antigen receptor; CTLA-4, Cytotoxic T-lymphocyte-associated protein 4; PD-1, Programmed cell death protein 1; PD-L1, Programmed death ligand 1; scFv, Single-chain variable fragment.

Unlike transferred TILs, CAR-T and TCR-T cells are limited in their ability to recognize a single peptide despite the heterogeneity of antigens in solid tumors. The outgrowth of tumor cells expressing irrelevant antigens is one of the main causes of relapse following ACT. Hence, a number of groups are dedicated to combining cellular therapy with other treatments to stimulate host immunity and invigorate engineered cells. In addition to immunoregulatory cytokines, adoptive cells can also be engineered to express a bispecific T cell engager (BiTE). Bispecific T-cell engagers (BiTEs) are engineered molecules that simultaneously bind to tumor antigens and CD3 on T cells, thereby physically bridging T cells to cancer cells and promoting T cell-mediated killing. When incorporated into adoptive cell platforms, BiTE secretion can also help recruit bystander endogenous T cells to broaden the anti-tumor response and reduce antigen escape-mediated relapse (Fig. 3).^96^^,^^97^ Choi et al^96^ designed a CAR-T specific for EGFRvIII (the most common oncogenic deletion mutant of *EGFR*) that secretes a BiTE against EGFR. In this case, EGFR-expressing tumor cells that escape recognition and clearance by EGFRvIII CAR-T cells would be compelled to other T cells via the engager, increasing the likelihood of being killed. In an investigator-initiated, open-label study,^98^ CARv3-TEAM-E T cells contributed to dramatic and rapid tumor regression in recurrent glioblastoma. Apart from mobilizing bystander T cells from the host, researchers^99^^,^^100^ are using oncolytic viruses to deliver CAR targets to solid tumors. By forcing tumor cells to express a non-signaling, truncated CD19 protein, CD19-CAR-T cells can recognize and eradicate a broader range of tumor cells, accompanied by the release of viruses from dying tumor cells, resulting in a waterfall effect^99^ (Fig. 3).

### Simplifying the manufacturing process and optimizing accessibility

ACT is known for its complex production process, long manufacturing cycle, and high cost. Diverse approaches are underway to increase the accessibility of ACT.

Allogeneic adoptive cells derived from healthy donors surpass autogenous cells in terms of culture stability and greater potency.^101^^,^^102^ The off-the-shelf character greatly reduces preparation time before being applied to the patient, which is crucial for cancer treatment (Fig. 4). Nonetheless, the immune system recognizes “self” from “non-self”, raising a dilemma for the tolerance of the transferred allogeneic cells in the recipient. Allogeneic T cell therapies must overcome the challenge of graft-versus-host disease (GVHD) caused by transferred cells to achieve broad use. Deletion of the MHC I molecule on donor cells may help evade recognition of the receiver’s T cells but can lead to increased killing by NK cells. Scientists from the Peking-Tsinghua Center for Life Sciences^103^ offered a solution by modifying the glycan profiles of the donor T cells. Another team took advantage of CD47, a commonly known “don’t eat me signal”, in CD19-CAR-T therapy.^104^ To protect the host from GVHD, two phase 1 studies^105^ enrolled patients to receive UCART19, a donor-derived anti-CD19 CAR-T product lacking a TCR.

**Fig. 4:**
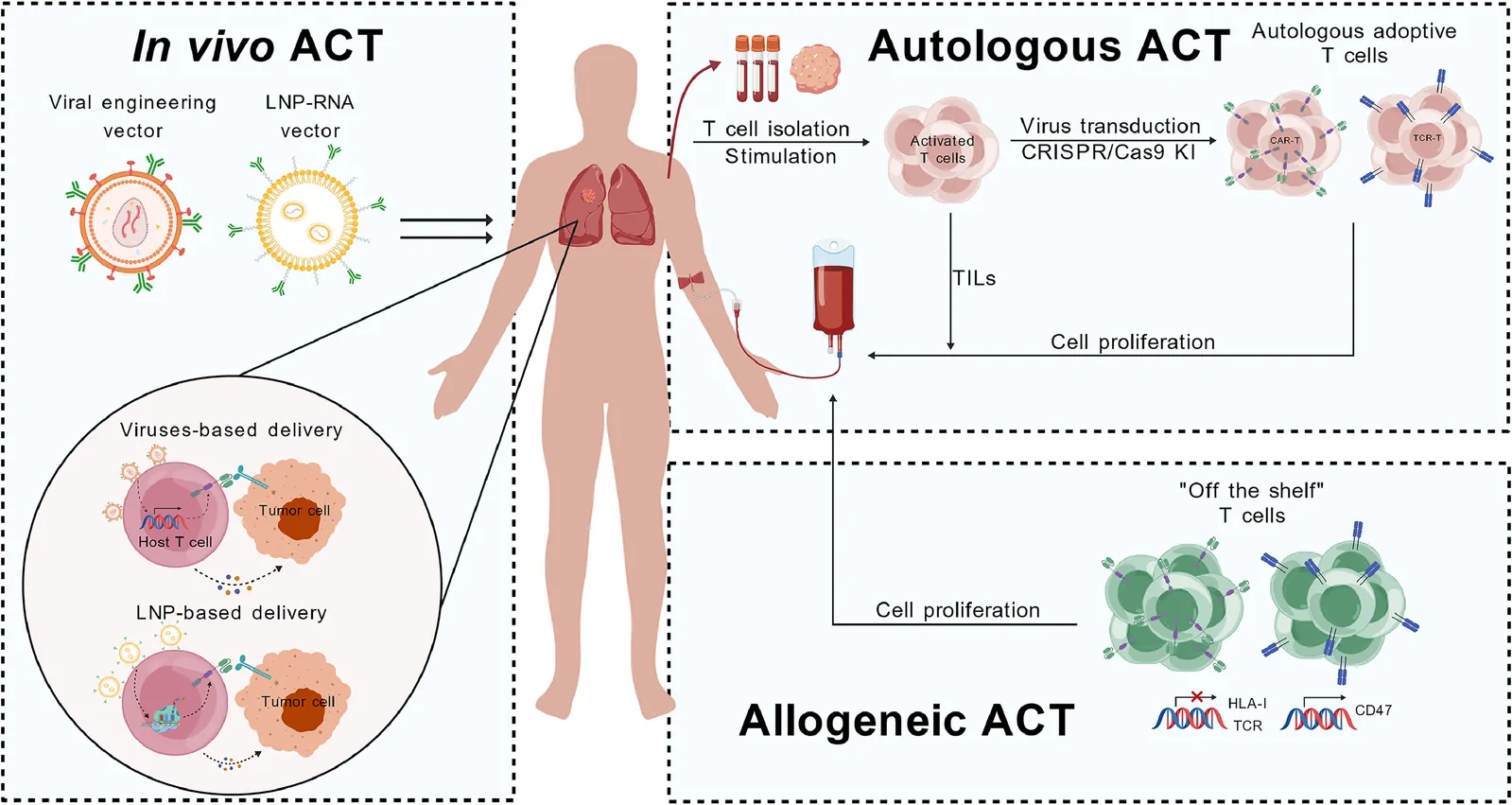
Current sources of adoptive cells and the principle of *in vivo* engineering ACT approaches. Tumor-infiltrating T cells are isolated from tumor tissue, whereas CAR-T and TCR-T cells are derived from PBMC. Allogeneic adoptive cells are derived from healthy donors and are ready for use. As for *in vivo* ACT, viral engineering and LNP-RNA are two main vectors for gene delivery. The vectors delivered *in vivo* edit host T cells to express tumor antigen-recognition receptors and facilitate anti-tumor immunity. Created with BioGDP.com. ACT, Adoptive cell therapy; CAR-T, Chimeric antigen receptor T-cell therapy; CRISPR/Cas9, Clustered regularly interspaced short palindromic repeats/CRISPR-associated protein 9; HLA, Human leukocyte antigen; KI, Knock-in; LNP, lipid nanoparticle; PBMC, Peripheral blood mononuclear cells; TCR, T cell receptor; TILs, Tumor-infiltrating lymphocytes.

Apart from the resources of adoptive cells, manufacturing methods have also been optimized for safer and more efficient output. Forced gene expression in T cells *ex vivo* commonly relies on randomly integrating vectors,^106^ mostly retroviruses or lentiviruses. The random integration of viral target genes into the host genome can lead to oncogenic transformation, clonal expansion, and gene silencing. Hence, researchers are developing next-generation targeted gene delivery methods based on the CRISPR/CRISPR-associated protein 9 (Cas9) technology. Zhang et al^107^ utilized an adeno-associated virus (AAV) vector as a template, combined with a non-viral insertion strategy, to develop a CAR-T product with high safety and effectiveness for treating B cell non-Hodgkin lymphoma. In addition to the CAR element, investigators have attempted to insert two chains of a neoantigen-specific TCR into the T cell receptor alpha constant (TRAC) gene locus of circulating T cells isolated from patients.^108^ This product was administered in a phase I clinical trial (NCT03970382) to evaluate its safety and potency for refractory solid cancers, including lung cancer.^108^

The methods discussed above rely on *ex vivo* engineering of adoptive cells before transfer to patients. Nevertheless, *in vivo* CAR-T cell therapy offers a promising future for directly manipulating immune cells using scalable, off-the-shelf biomedical products. Viruses and lipid nanoparticles (LNP)-based RNA delivery are the two major platforms for *in vivo* engineering. There are many *in vivo* CAR candidates with promising preclinical results that have entered clinical trials for hematological malignancies and solid tumors^109^, ^110^, ^111^, ^112^ (Fig. 4).

### Exploiting other immune candidates beyond traditional T cells

The immune system consists of two unique but inseparable parts: the adaptive system, which is responsible for specific antigen recognition and durable protection; and the innate system, which plays a pivotal role in immune surveillance without specificity and immune memory. Currently, cells from the innate immune system are considered promising candidates for adoptive therapy and have been evaluated in preclinical and clinical studies.

NK cells derived from cord blood have been transduced with anti-CD19 CAR and interleukin-15 to enhance their *in vivo* expansion and persistence.^113^^,^^114^ The administration of CAR-NK cells contributed to a response rate of 73% in 11 patients with CD19-positive lymphoid tumors without inducing cytokine release syndrome, neurotoxicity, or other inflammatory side effects.^114^ Compared with traditional T-cell therapy, NK-cell therapy offers the following advantages: (1) greater safety, particularly lower risks of GVHD, cytokine release syndrome, and neurotoxicity; (2) greater suitability for off-the-shelf production, enabling faster manufacturing, greater accessibility, and greater potential for universal application; (3) independence from classic HLA restrictions, allowing the recognition of tumors with downregulated HLA-I or T-cell evasion; (4) wider applicability, making it particularly suitable for the elderly, frail patients, those with poor T-cell quality, and patients requiring rapid treatment. However, it faces challenges such as poor persistence *in vivo* and insufficient expansion; difficulties with homing, infiltration, and survival in solid tumors; and problems with CAR viral transduction. Consequently, NK-cell therapy is not widely used in clinical practice.^115^

Macrophages have also intrigued researchers owing to their abundant infiltration in tumors, their immunomodulatory properties, and their ability to phagocytose tumor cells directly. In 2020, Klichinsky et al^116^ reported that genetically engineered human macrophages with CARs were manipulated into a pro-inflammatory (M1) phenotype and contributed to antigen-specific phagocytosis in solid tumors. The second generation of CAR-macrophages (CAR-M), originating from pluripotent stem cells^117^ and with an elaborately modified intracellular domain, showed an enhanced anti-tumor effect compared to the first generation. Reiss et al^118^ conducted a first-in-human, phase 1, multicenter clinical trial evaluating the safety and efficiency of autologous anti-HER2 CAR-macrophages for advanced HER2-expressing solid tumors. This product reduces circulating tumor DNA (ctDNA) levels and is associated with SD as the best overall response (BOR) in a subset of patients without dose-limiting toxicities (DLT) (NCT04660929). Compared with traditional CAR-T therapy, CAR-M therapy offers the following advantages: (1) Easier access to tumor tissue because macrophages naturally infiltrate tumor tissue, whereas CAR-T cells often face challenges in solid tumors, such as difficulty in penetrating the tumor, poor retention, and low survival rates; (2) CAR-M can not only directly phagocytose target cells, but also process and present tumor antigens, further activating the innate immune system and eliciting a broader and more sustained antitumor immune response; (3) CAR-M can reshape the immunosuppressive tumor microenvironment. However, the following limitations exist: (1) Limited clinical evidence. CAR-T therapy already has multiple established success stories in hematologic malignancies, whereas CAR-M therapy is still in the early stages, and further data are needed to support its true clinical benefits. (2) Macrophages are not as easily expanded, transduced, or maintained long term as T cells, and their production process is more complex. (3) Macrophages may be “educated” within the tumor microenvironment, leading to reduced antitumor activity, and their persistence may be inferior to that of CAR-T.^119^^,^^120^

In addition, as the most abundant white blood cells in the circulation, neutrophils have also been engineered to express CAR molecules.^121^^,^^122^ CAR-neutrophils help deliver nanodrugs specifically to the TME in glioblastoma (GBM) beyond direct cytotoxicity.^122^ However, the short lifespan of neutrophils prevents long-term tumor control and immune surveillance with these cells, requiring novel strategies or therapy combinations.

## Conclusion and perspectives

We discuss various approaches to achieving both potent anti-tumor activity and safety in healthy tissues in ACTs. Currently, signal transduction domains, immune regulatory transcription factors, cytokines, and tumor-associated targets, as well as numerous types of transferred cells, are under evaluation in both laboratory and clinical trials. Apart from the ongoing biological engineering attempts, it is of great importance to elucidate the characteristics of adoptive cells that best suit the tumor circumstances and help sustain long-term protection, and how adoptive immune cells interact with and influence the host.

ACT has brought cancer treatment into a new and promising era. The glory won from hematological malignancies fueled the confidence of clinical doctors and basic science researchers. Nevertheless, the complex cellular components, dense structure, and unpredictable antigen targets present difficulties for the use of adoptive cells in lung cancer. However, with the development of synthetic biology and artificial intelligence (AI), more tools are emerging for engineering and manipulating adoptive cells. A major priority is the mass production of off-the-shelf universal CAR-T cells. Advances in gene editing technologies such as CRISPR/Cas9 systems and base editing have made it increasingly feasible to eliminate endogenous TCR and HLA molecules, thereby reducing the risks of GVHD and host-mediated rejection. For example, knocking out signal peptide peptidase like 3 (SPPL3) using gene editing technology can alter glycosylation modifications on the surface of T cells, thereby enhancing the persistence of CAR-T cells in patients.^103^ In parallel, synthetic biology has enabled the design of CAR-T cells with programmable features, including logic-gated activation, inducible cytokine release, enhanced resistance to immunosuppressive signals, and controllable safety switches. These innovations could support the transition from patient-specific manufacturing to standardized allogeneic products, which would substantially reduce the production time, cost, and batch-to-batch variability, making CAR-T therapy more accessible in routine clinical settings. Another key direction is AI-guided personalized CAR design. One of the central barriers in solid tumor treatment is antigen heterogeneity and the lack of truly tumor-specific targets. AI-based integration of multiomics data, single-cell transcriptomics, spatial profiling, and proteomic datasets can accelerate the identification of antigen combinations that maximize tumor specificity while minimizing off-tumor toxicity. In the short to medium term, this approach may enable the rational design of personalized CAR constructs as well as adaptive treatment strategies tailored to the antigenic architecture of individual tumors.^123^ As the characteristics and components of cells in tumor immunology are unveiled,^124^ we look forward to adoptive cellular products with both potency and safety for lung cancer treatment.

## Funding

This work was supported by the Jiebang Guashuai Program from The Second Affiliated Hospital of Army Medical University (No. 2023XKRC002 to Bo Zhu).

## CRediT authorship contribution statement

**Jingyi Li:** Writing – original draft, Visualization, Validation, Investigation. **Chan Tong:** Writing – original draft, Investigation. **Yongchang Chen:** Writing – original draft, Investigation. **Bo Zhu:** Writing – review & editing, Supervision, Project administration, Funding acquisition, Conceptualization.

## Declaration of competing interests

The authors declare that they have no known competing financial interests or personal relationships that could have appeared to influence the work reported in this paper.

## Appendix Supplementary materials

Image, application 1

## References

[1] Sung H., Ferlay J., Siegel R.L. (2021). Global Cancer Statistics 2020: GLOBOCAN estimates of incidence and mortality worldwide for 36 cancers in 185 countries. CA Cancer J Clin.

[2] Ricotti A., Sciannameo V., Balzi W. (2021). Incidence and prevalence analysis of non-small-cell and small-cell lung cancer using administrative data. Int J Environ Res Public Health.

[3] Allemani C., Matsuda T., Di Carlo V. (2018). Global surveillance of trends in cancer survival 2000-14 (CONCORD-3): analysis of individual records for 37 513 025 patients diagnosed with one of 18 cancers from 322 population-based registries in 71 countries. Lancet.

[4] Novello S., Kowalski D.M., Luft A. (2023). Pembrolizumab plus chemotherapy in squamous non-small-cell lung cancer: 5-year update of the phase III KEYNOTE-407 study. J Clin Oncol.

[5] Garassino M.C., Gadgeel S., Speranza G. (2023). Pembrolizumab plus pemetrexed and platinum in nonsquamous non-small-cell lung cancer: 5-year outcomes from the phase 3 KEYNOTE-189 study. J Clin Oncol.

[6] Tang H., Wang Y., Chlewicki L.K. (2016). Facilitating T cell infiltration in tumor microenvironment overcomes resistance to PD-L1 blockade. Cancer Cell.

[7] Yost K.E., Satpathy A.T., Wells D.K. (2019). Clonal replacement of tumor-specific T cells following PD-1 blockade. Nat Med.

[8] Guo X., Zhang Y., Zheng L. (2018). Global characterization of T cells in non-small-cell lung cancer by single-cell sequencing. Nat Med.

[9] Miller B.C., Sen D.R., Al Abosy R. (2019). Subsets of exhausted CD8+ T cells differentially mediate tumor control and respond to checkpoint blockade. Nat Immunol.

[10] Schoenfeld A.J., Lee S.M., Doger de Spéville B. (2024). Lifileucel, an autologous tumor-infiltrating lymphocyte monotherapy, in patients with advanced non-small cell lung cancer resistant to immune checkpoint inhibitors. Cancer Discov.

[11] Wei X., Lai Y., Li J. (2017). PSCA and MUC1 in non-small-cell lung cancer as targets of chimeric antigen receptor T cells. Oncoimmunology.

[12] Kiesgen S., Chicaybam L., Chintala N.K., Adusumilli P.S. (2018). Chimeric antigen receptor (CAR) T-cell therapy for thoracic malignancies. J Thorac Oncol.

[13] Baulu E., Gardet C., Chuvin N., Depil S. (2023). TCR-engineered T cell therapy in solid tumors: state of the art and perspectives. Sci Adv.

[14] Xia Y., Tian X., Wang J. (2018). Treatment of metastatic non-small cell lung cancer with NY-ESO-1 specific TCR engineered-T cells in a phase I clinical trial: a case report. Oncol Lett.

[15] Zhong R., Chu T., Xiong L. (2025). Efficacy and safety of autologous CIK cell therapy plus Toripalimab with or without chemotherapy in advanced NSCLC: a phase II study. Int J Cancer.

[16] Hong G., Chen X., Sun X. (2019). Effect of autologous NK cell immunotherapy on advanced lung adenocarcinoma with EGFR mutations. Precis Clin Med.

[17] Kershaw M.H., Westwood J.A., Darcy P.K. (2013). Gene-engineered T cells for cancer therapy. Nat Rev Cancer.

[18] Zhao S., Zhao H., Yang W., Zhang L. (2025). The next generation of immunotherapies for lung cancers. Nat Rev Clin Oncol.

[19] Lu J., Jiang G. (2022). The journey of CAR-T therapy in hematological malignancies. Mol Cancer.

[20] Julve M., Lythgoe M.P., Larkin J., Furness A.J.S. (2024). Lifileucel: the first cellular therapy approved for solid tumours. Trends Cancer.

[21] Norberg S.M., Hinrichs C.S. (2023). Engineered T cell therapy for viral and non-viral epithelial cancers. Cancer Cell.

[22] Zhang P., Zhang G., Wan X. (2023). Challenges and new technologies in adoptive cell therapy. J Hematol Oncol.

[23] Hu W., Bian Y., Ji H. (2024). TIL therapy in lung cancer: current progress and perspectives. Adv Sci (Weinh).

[24] Wang S., Sun J., Chen K. (2021). Perspectives of tumor-infiltrating lymphocyte treatment in solid tumors. BMC Med.

[25] Rosenberg S.A., Packard B.S., Aebersold P.M. (1988). Use of tumor-infiltrating lymphocytes and interleukin-2 in the immunotherapy of patients with metastatic melanoma. A preliminary report. N Engl J Med.

[26] Rosenberg S.A., Yang J.C., Sherry R.M. (2011). Durable complete responses in heavily pretreated patients with metastatic melanoma using T-cell transfer immunotherapy. Clin Cancer Res.

[27] Besser M.J., Shapira-Frommer R., Itzhaki O. (2013). Adoptive transfer of tumor-infiltrating lymphocytes in patients with metastatic melanoma: intent-to-treat analysis and efficacy after failure to prior immunotherapies. Clin Cancer Res.

[28] Goff S.L., Dudley M.E., Citrin D.E. (2016). Randomized, prospective evaluation comparing intensity of lymphodepletion before adoptive transfer of tumor-infiltrating lymphocytes for patients with metastatic melanoma. J Clin Oncol.

[29] Andersen R., Donia M., Ellebaek E. (2016). Long-lasting complete responses in patients with metastatic melanoma after adoptive cell therapy with tumor-infiltrating lymphocytes and an attenuated IL2 regimen. Clin Cancer Res.

[30] Stevanović S., Helman S.R., Wunderlich J.R. (2019). A phase II study of tumor-infiltrating lymphocyte therapy for human Papillomavirus-associated epithelial cancers. Clin Cancer Res.

[31] Tran E., Robbins P.F., Lu Y.C. (2016). T-cell transfer therapy targeting mutant KRAS in cancer. N Engl J Med.

[32] Zacharakis N., Chinnasamy H., Black M. (2018). Immune recognition of somatic mutations leading to complete durable regression in metastatic breast cancer. Nat Med.

[33] Ratto G.B., Zino P., Mirabelli S. (1996). A randomized trial of adoptive immunotherapy with tumor-infiltrating lymphocytes and interleukin-2 versus standard therapy in the postoperative treatment of resected nonsmall cell lung carcinoma. Cancer.

[34] Creelan B.C., Wang C., Teer J.K. (2021). Tumor-infiltrating lymphocyte treatment for anti-PD-1-resistant metastatic lung cancer: a phase 1 trial. Nat Med.

[35] Sarnaik A.A., Hamid O., Khushalani N.I. (2021). Lifileucel, a tumor-infiltrating lymphocyte therapy, in metastatic melanoma. J Clin Oncol.

[36] Chesney J., Lewis K.D., Kluger H. (2022). Efficacy and safety of lifileucel, a one-time autologous tumor-infiltrating lymphocyte (TIL) cell therapy, in patients with advanced melanoma after progression on immune checkpoint inhibitors and targeted therapies: pooled analysis of consecutive cohorts of the C-144-01 study. J Immunother Cancer.

[37] Titov A., Zmievskaya E., Ganeeva I. (2021). Adoptive immunotherapy beyond CAR T-cells. Cancers.

[38] Monberg T.J., Borch T.H., Svane I.M., Donia M. (2023). TIL therapy: facts and hopes. Clin Cancer Res.

[39] Barr T., Ma S., Li Z., Yu J. (2024). Recent advances and remaining challenges in lung cancer therapy. Chin Med J (Engl).

[40] Yao X., Ahmadzadeh M., Lu Y.C. (2012). Levels of peripheral CD4(+)FoxP3(+) regulatory T cells are negatively associated with clinical response to adoptive immunotherapy of human cancer. Blood.

[41] Shah K., Al-Haidari A., Sun J., Kazi J.U. (2021). T cell receptor (TCR) signaling in health and disease. Signal Transduct Target Ther.

[42] Xu R., Du S., Zhu J., Meng F., Liu B. (2022). Neoantigen-targeted TCR-T cell therapy for solid tumors: how far from clinical application. Cancer Lett.

[43] Shafer P., Kelly L.M., Hoyos V. (2022). Cancer therapy with TCR-engineered T cells: current strategies, challenges, and prospects. Front Immunol.

[44] Chandran S.S., Klebanoff C.A. (2019). T cell receptor-based cancer immunotherapy: emerging efficacy and pathways of resistance. Immunol Rev.

[45] Kim S.H., Lee S., Lee C.H. (2009). Expression of cancer-testis antigens MAGE-A3/6 and NY-ESO-1 in non-small-cell lung carcinomas and their relationship with immune cell infiltration. Lung.

[46] Gjerstorff M.F., Pøhl M., Olsen K.E., Ditzel H.J. (2013). Analysis of GAGE, NY-ESO-1 and SP17 cancer/testis antigen expression in early stage non-small cell lung carcinoma. BMC Cancer.

[47] Blumenschein G.R., Devarakonda S., Johnson M. (2022). Phase I clinical trial evaluating the safety and efficacy of ADP-A2M10 SPEAR T cells in patients with MAGE-A10+ advanced non-small cell lung cancer. J ImmunoTher Cancer.

[48] D’Angelo S.P., Araujo D.M., Abdul Razak A.R. (2024). Afamitresgene autoleucel for advanced synovial sarcoma and myxoid round cell liposarcoma (SPEARHEAD-1): an international, open-label, phase 2 trial. Lancet.

[49] Keam S.J. (2024). Afamitresgene autoleucel: first approval. Mol Diagn Ther.

[50] Garcia V.M., Calvo E., Asch A. (2023). 1019O clinical and translational data from the phase I SURPASS trial of ADP-A2M4CD8 T cell receptor (TCR) T cell therapy alone or combined with nivolumab in solid tumors. Ann Oncol.

[51] Albelda S.M. (2024). CAR T cell therapy for patients with solid tumours: key lessons to learn and unlearn. Nat Rev Clin Oncol.

[52] Ma S., Li X., Wang X. (2019). Current progress in CAR-T cell therapy for solid tumors. Int J Biol Sci.

[53] Sterner R.C., Sterner R.M. (2021). CAR-T cell therapy: current limitations and potential strategies. Blood Cancer J.

[54] Caliendo F., Dukhinova M., Siciliano V. (2019). Engineered cell-based therapeutics: synthetic biology meets immunology. Front Bioeng Biotechnol.

[55] Ying Z., Huang X.F., Xiang X. (2019). A safe and potent anti-CD19 CAR T cell therapy. Nat Med.

[56] Xiao B.F., Zhang J.T., Zhu Y.G. (2021). Chimeric antigen receptor T-cell therapy in lung cancer: potential and challenges. Front Immunol.

[57] Feng K., Guo Y., Dai H. (2016). Chimeric antigen receptor-modified T cells for the immunotherapy of patients with EGFR-expressing advanced relapsed/refractory non-small cell lung cancer. Sci China Life Sci.

[58] Zhang Y., Zhang Z., Ding Y. (2021). Phase I clinical trial of EGFR-specific CAR-T cells generated by the piggyBac transposon system in advanced relapsed/refractory non-small cell lung cancer patients. J Cancer Res Clin Oncol.

[59] Kachala S.S., Bograd A.J., Villena-Vargas J. (2014). Mesothelin overexpression is a marker of tumor aggressiveness and is associated with reduced recurrence-free and overall survival in early-stage lung adenocarcinoma. Clin Cancer Res.

[60] Adusumilli P.S., Zauderer M.G., Rivière I. (2021). A phase I trial of regional mesothelin-targeted CAR T-cell therapy in patients with malignant pleural disease, in combination with the anti-PD-1 agent pembrolizumab. Cancer Discov.

[61] Lin Y., Chen S., Zhong S., An H., Yin H., McGowan E. (2019). 35O - phase I clinical trial of PD-1 knockout anti-MUC1 CAR-T cells in the treatment of patients with non-small cell lung cancer. Ann Oncol.

[62] Sharma S.K., Pourat J., Abdel-Atti D. (2017). Noninvasive interrogation of DLL3 expression in metastatic small cell lung cancer. Cancer Res.

[63] Flugel C.L., Majzner R.G., Krenciute G. (2023). Overcoming on-target, off-tumour toxicity of CAR T cell therapy for solid tumours. Nat Rev Clin Oncol.

[64] Ma H.Y., Das J., Prendergast C. (2023). Advances in CAR T cell therapy for non-small cell lung cancer. Curr Issues Mol Biol.

[65] Chen J., Qiu S., Li W. (2023). Tuning charge density of chimeric antigen receptor optimizes tonic signaling and CAR-T cell fitness. Cell Res.

[66] Long A.H., Haso W.M., Shern J.F. (2015). 4-1BB costimulation ameliorates T cell exhaustion induced by tonic signaling of chimeric antigen receptors. Nat Med.

[67] Velasco Cárdenas R.M., Brandl S.M., Meléndez A.V. (2023). Harnessing CD3 diversity to optimize CAR T cells. Nat Immunol.

[68] Wu W., Zhou Q., Masubuchi T. (2020). Multiple signaling roles of CD3ε and its application in CAR-T cell therapy. Cell.

[69] Shukla D., Manne S., Jiang S., Ruella M., Wherry E.J., Riley J.L. (2025). Optimal pairing of binder and co-stimulatory domains improves dual CART cell efficacy. Mol Ther.

[70] Liu Y., Liu G., Wang J. (2021). Chimeric STAR receptors using TCR machinery mediate robust responses against solid tumors. Sci Transl Med.

[71] Huang D., Li Y., Rui W. (2024). TCR-mimicking STAR conveys superior sensitivity over CAR in targeting tumors with low-density neoantigens. Cell Rep.

[72] Chan J.D., Scheffler C.M., Munoz I. (2024). FOXO1 enhances CAR T cell stemness, metabolic fitness and efficacy. Nature.

[73] Doan A.E., Mueller K.P., Chen A.Y. (2024). FOXO1 is a master regulator of memory programming in CAR T cells. Nature.

[74] Chen J., López-Moyado I.F., Seo H. (2019). NR4A transcription factors limit CAR T cell function in solid tumours. Nature.

[75] Liu X., Wang Y., Lu H. (2019). Genome-wide analysis identifies NR4A1 as a key mediator of T cell dysfunction. Nature.

[76] Zhang J., Li J., Hou Y. (2024). Osr2 functions as a biomechanical checkpoint to aggravate CD8+ T cell exhaustion in tumor. Cell.

[77] Allen G.M., Frankel N.W., Reddy N.R. (2022). Synthetic cytokine circuits that drive T cells into immune-excluded tumors. Science.

[78] Adachi K., Kano Y., Nagai T., Okuyama N., Sakoda Y., Tamada K. (2018). IL-7 and CCL19 expression in CAR-T cells improves immune cell infiltration and CAR-T cell survival in the tumor. Nat Biotechnol.

[79] Pang N., Shi J., Qin L. (2021). IL-7 and CCL19-secreting CAR-T cell therapy for tumors with positive glypican-3 or mesothelin. J Hematol Oncol.

[80] Steffin D., Ghatwai N., Montalbano A. (2025). Interleukin-15-armoured GPC3 CAR T cells for patients with solid cancers. Nature.

[81] Zhao Y., Chen J., Andreatta M. (2024). IL-10-expressing CAR T cells resist dysfunction and mediate durable clearance of solid tumors and metastases. Nat Biotechnol.

[82] Guo Y., Xie Y.Q., Gao M. (2021). Metabolic reprogramming of terminally exhausted CD8+ T cells by IL-10 enhances anti-tumor immunity. Nat Immunol.

[83] Zhang L., Kerkar S.P., Yu Z. (2011). Improving adoptive T cell therapy by targeting and controlling IL-12 expression to the tumor environment. Mol Ther.

[84] Jaspers J.E., Khan J.F., Godfrey W.D. (2023). IL-18-secreting CAR T cells targeting DLL3 are highly effective in small cell lung cancer models. J Clin Invest.

[85] Lai J., Mardiana S., House I.G. (2020). Adoptive cellular therapy with T cells expressing the dendritic cell growth factor Flt3L drives epitope spreading and anti-tumor immunity. Nat Immunol.

[86] Hyrenius-Wittsten A., Su Y., Park M. (2021). SynNotch CAR circuits enhance solid tumor recognition and promote persistent anti-tumor activity in mouse models. Sci Transl Med.

[87] Roybal K.T., Williams J.Z., Morsut L. (2016). Engineering T cells with customized therapeutic response programs using synthetic notch receptors. Cell.

[88] Simic M.S., Watchmaker P.B., Gupta S. (2024). Programming tissue-sensing T cells that deliver therapies to the brain. Science.

[89] Chen A.X.Y., Yap K.M., Kim J.S. (2025). Rewiring endogenous genes in CAR T cells for tumour-restricted payload delivery. Nature.

[90] Bagley S.J., Logun M., Fraietta J.A. (2024). Intrathecal bivalent CAR T cells targeting EGFR and IL13Rα2 in recurrent glioblastoma: phase 1 trial interim results. Nat Med.

[91] Huber F., Arnaud M., Stevenson B.J. (2025). A comprehensive proteogenomic pipeline for neoantigen discovery to advance personalized cancer immunotherapy. Nat Biotechnol.

[92] Grosser R., Cherkassky L., Chintala N., Adusumilli P.S. (2019). Combination immunotherapy with CAR T cells and checkpoint blockade for the treatment of solid tumors. Cancer Cell.

[93] Cherkassky L., Morello A., Villena-Vargas J. (2016). Human CAR T cells with cell-intrinsic PD-1 checkpoint blockade resist tumor-mediated inhibition. J Clin Invest.

[94] Agarwal S., Aznar M.A., Rech A.J. (2023). Deletion of the inhibitory co-receptor CTLA-4 enhances and invigorates chimeric antigen receptor T cells. Immunity.

[95] Rafiq S., Yeku O.O., Jackson H.J. (2018). Targeted delivery of a PD-1-blocking scFv by CAR-T cells enhances anti-tumor efficacy *in vivo*. Nat Biotechnol.

[96] Choi B.D., Yu X., Castano A.P. (2019). CAR-T cells secreting BiTEs circumvent antigen escape without detectable toxicity. Nat Biotechnol.

[97] Wing A., Fajardo C.A., Posey A.D.Jr (2018). Improving CART-cell therapy of solid tumors with oncolytic virus-driven production of a bispecific T-cell engager. Cancer Immunol Res.

[98] Choi B.D., Gerstner E.R., Frigault M.J. (2024). Intraventricular CARv3-TEAM-E T cells in recurrent glioblastoma. N Engl J Med.

[99] Park A.K., Fong Y., Kim S.I. (2020). Effective combination immunotherapy using oncolytic viruses to deliver CAR targets to solid tumors. Sci Transl Med.

[100] Guedan S., Alemany R. (2018). CAR-T cells and oncolytic viruses: joining forces to overcome the solid tumor challenge. Front Immunol.

[101] Wang X., Wu X., Tan B. (2024). Allogeneic CD19-targeted CAR-T therapy in patients with severe myositis and systemic sclerosis. Cell.

[102] Pal S.K., Tran B., Haanen JBAG (2024). CD70-targeted allogeneic CAR T-cell therapy for advanced clear cell renal cell carcinoma. Cancer Discov.

[103] Wu Z., Shi J., Lamao Q. (2025). Glycan shielding enables TCR-sufficient allogeneic CAR-T therapy. Cell.

[104] Hu X., Manner K., DeJesus R. (2023). Hypoimmune anti-CD19 chimeric antigen receptor T cells provide lasting tumor control in fully immunocompetent allogeneic humanized mice. Nat Commun.

[105] Benjamin R., Graham C., Yallop D. (2020). Genome-edited, donor-derived allogeneic anti-CD19 chimeric antigen receptor T cells in paediatric and adult B-cell acute lymphoblastic leukaemia: results of two phase 1 studies. Lancet.

[106] Eyquem J., Mansilla-Soto J., Giavridis T. (2017). Targeting a CAR to the TRAC locus with CRISPR/Cas9 enhances tumour rejection. Nature.

[107] Zhang J., Hu Y., Yang J. (2022). Non-viral, specifically targeted CAR-T cells achieve high safety and efficacy in B-NHL. Nature.

[108] Foy S.P., Jacoby K., Bota D.A. (2023). Non-viral precision T cell receptor replacement for personalized cell therapy. Nature.

[109] Ramos C.A., Rouce R., Robertson C.S. (2018). *In vivo* fate and activity of second- versus third-generation CD19-specific CAR-T cells in B Cell non-Hodgkin’s lymphomas. Mol Ther.

[110] Xu J., Liu L., Parone P. (2025). *In-vivo* B-cell maturation antigen CAR T-cell therapy for relapsed or refractory multiple myeloma. Lancet.

[111] Bot A., Scharenberg A., Friedman K. (2026). *In vivo* chimeric antigen receptor (CAR)-T cell therapy. Nat Rev Drug Discov.

[112] Rodriguez J., De Iaco A., Rottman J.B. (2024). Targeted lipid nanoparticle delivery of an RNA gene writer *in vivo* enables generation of CAR-T cells in a humanized house model. Blood.

[113] Liu E., Tong Y., Dotti G. (2018). Cord blood NK cells engineered to express IL-15 and a CD19-targeted CAR show long-term persistence and potent anti-tumor activity. Leukemia.

[114] Liu E., Marin D., Banerjee P. (2020). Use of CAR-transduced natural killer cells in CD19-positive lymphoid tumors. N Engl J Med.

[115] Peng L., Sferruzza G., Yang L., Zhou L., Chen S. (2024). CAR-T and CAR-NK as cellular cancer immunotherapy for solid tumors. Cell Mol Immunol.

[116] Klichinsky M., Ruella M., Shestova O. (2020). Human chimeric antigen receptor macrophages for cancer immunotherapy. Nat Biotechnol.

[117] Lei A., Yu H., Lu S. (2024). A second-generation M1-polarized CAR macrophage with anti-tumor efficacy. Nat Immunol.

[118] Reiss K.A., Angelos M.G., Dees E.C. (2025). CAR-macrophage therapy for HER2-overexpressing advanced solid tumors: a phase 1 trial. Nat Med.

[119] Look T., Sankowski R., Bouzereau M. (2025). CAR T cells, CAR NK cells, and CAR macrophages exhibit distinct traits in glioma models but are similarly enhanced when combined with cytokines. Cell Rep Med.

[120] Wang M., Qin Z., Bian X.W., Shi Y. (2025). Harnessing chimeric antigen receptor macrophages against solid tumors. Cancer Commun.

[121] Chang Y., Syahirah R., Wang X. (2022). Engineering chimeric antigen receptor neutrophils from human pluripotent stem cells for targeted cancer immunotherapy. Cell Rep.

[122] Chang Y., Cai X., Syahirah R. (2023). CAR-neutrophil mediated delivery of tumor-microenvironment responsive nanodrugs for glioblastoma chemo-immunotherapy. Nat Commun.

[123] Chow A., Chu H., Li R. (2025). Sequence and structural determinants of efficacious *de novo* chimeric antigen receptors. bioRxiv.

[124] Zhou Q., Sun F., Wang X. (2025). Chimeric antigen receptor-T cell therapy for lung cancer: the tumor microenvironment bottleneck and remedies to circumvent it. Chin Med J Pulm Crit Care Med.

