# Supplementary material for: Adoptive cell therapy in lung cancer: progress, challenges, and prospects
Source: Chin Med J Pulm Crit Care Med. 2026 Sep 7;4(3):235–49. doi: 10.1016/j.pccm.2026.08.002 (PMC13649508; doi:10.1016/j.pccm.2026.08.002)
Supplement: Supplementary file 1 [file mmc1.docx]

**Supplementary Table 1: Trial-level data used for exploratory cross-platform comparison of published adoptive T cell therapy efficacy in lung cancer.**

| **NCT No.** | **ACT type** | **Histology**  **(NSCLC/SCLC)** | **Treatment line**  **(first line/later line)** | **ORR *n*/*N*** | **DCR *n*/*N*** | **Reference** |
| --- | --- | --- | --- | --- | --- | --- |
| NCT03215810 | TIL | NSCLC | Later line | 3/13 | NR | DOI: 10.1038/s41591-021-01462-y |
| NCT03645928  (Cohort 3A) | TIL | NSCLC | First line | 10/22 | NR | https://doi.org/10.1136/jitc-2024-SITC2024.1488 |
| NCT03645928  (Cohort 3B) | TIL | NSCLC | Later line | 6/28 | 18/28 | DOI: 10.1158/2159-8290.CD-23-1334 |
| NCT04614103 | TIL | NSCLC | Later line | 10/39 | 28/39 | https://ir.iovance.com/news-events/events-presentations. |
| NCT02592577 | TCR-T | NSCLC | Later line | 1/11 | 5/11 | DOI: 10.1136/jitc-2021-003581 |
| NCT04032847 | TCR-T | NSCLC | Later line | 2/27 | NR | https://clinicaltrials.gov/study/NCT04032847?term=NCT04032847&viewType=Card&rank=1&tab=results#outcome-measures |
| NCT02588612 | TCR-T | NSCLC | Later line | 1/5 | 1/5 | DOI: 10.1158/1078-0432.CCR-24-1591 |
| NCT01869166 | CAR-T | NSCLC | Later line | 2/11 | 7/11 | DOI: 10.1007/s11427-016-5023-8 |
| NCT03182816 | CAR-T | NSCLC | Later line | 1/9 | 7/9 | DOI: 10.1007/s00432-021-03613-7 |
| NCT03392064 | CAR-T | SCLC | Later line | 1/4 | 3/4 | DOI:10.1200/JCO.2025.43.16_suppl.e20561 |
| NCT02706392 | CAR-T | NSCLC | Later line | 1/8 | NR | DOI:10.1200/JCO.2025.43.16_suppl.e20561 |
| NCT03525782 | CAR-T | NSCLC | Later line | 0/20 | 11/20 | DOI:10.1200/JCO.2025.43.16_suppl.e20561 |

ACT, Adoptive cell therapy; CAR-T, Chimeric antigen receptor T-cell therapy; DCR, Disease control rate; NR: Not reported; NSCLC, Non-small cell lung cancer; ORR, Objective response rate; SCLC, Small cell lung cancer; TCR-T, T-cell receptor-engineered T-cell therapy; TIL, Tumor-infiltrating lymphocyte.
